# National and Provincial-Level Prevalence and Risk Factors of Carotid Atherosclerosis in Chinese Adults

**DOI:** 10.1001/jamanetworkopen.2023.51225

**Published:** 2024-01-11

**Authors:** Jingzhu Fu, Yuhan Deng, Yuan Ma, Sailimai Man, Xiaochen Yang, Canqing Yu, Jun Lv, Bo Wang, Liming Li

**Affiliations:** 1 Department of Epidemiology and Biostatistics, School of Public Health, Peking University, Beijing, China; 2 Meinian Public Health Institute, Peking University Health Science Center, Beijing, China; 3 Key Laboratory of Epidemiology of Major Diseases (Peking University), Ministry of Education, Beijing, China; 4 Meinian Institute of Health, Beijing, China; 5 Department of Social Medicine and Health Education, School of Public Health, Peking University, Beijing, China; 6 Chongqing Research Institute of Big Data, Peking University, Chongqing, China; 7 School of Population Medicine and Public Health, Chinese Academy of Medical Sciences & Peking Union Medical College, Beijing, China; 8 Peking University Center for Public Health and Epidemic Preparedness & Response, Beijing, China

## Abstract

**Importance:**

Epidemiologic studies on carotid atherosclerosis (CAS) based on nationwide ultrasonography measurements can contribute to understanding the future risk of cardiovascular diseases and identifying high-risk populations, thereby proposing more targeted prevention and treatment measures.

**Objectives:**

To estimate the prevalence of CAS within the general population of China and to investigate its distribution among populations with potential risk factors and variation across diverse geographic regions.

**Design, Setting, and Participants:**

This multicenter, population-based cross-sectional study used China’s largest health check-up chain database to study 10 733 975 individuals aged 20 years or older from all 31 provinces in China who underwent check-ups from January 1, 2017, to June 30, 2022.

**Main Outcomes and Measures:**

Carotid atherosclerosis was assessed and graded using ultrasonography as increased carotid intima-media thickness (cIMT), carotid plaque (CP), and carotid stenosis (CS). The overall and stratified prevalences were estimated among the general population and various subpopulations based on demographic characteristics, geographic regions, and cardiovascular disease risk factors. Mixed-effects regression models were used to analyze the risk factors for CAS.

**Results:**

Among 10 733 975 Chinese participants (mean [SD] age, 47.7 [13.4] years; 5 861 566 [54.6%] male), the estimated prevalences were 26.2% (95% CI, 25.0%-27.4%) for increased cIMT, 21.0% (95% CI, 19.8%-22.2%) for CP, and 0.56% (95% CI, 0.36%-0.76%) for CS. The prevalence of all CAS grades was higher among older adults (eg, increased cIMT: aged ≥80 years, 92.7%; 95% CI, 92.2%-93.3%), male participants (29.6%; 95% CI, 28.4%-30.7%), those residing in northern China (31.0%; 95% CI, 29.1%-32.9%), and those who had comorbid conditions, such as hypertension (50.8%; 95% CI, 49.7%-51.9%), diabetes (59.0%; 95% CI, 57.8%-60.1%), dyslipidemia (32.1%; 95% CI, 30.8%-33.3%), and metabolic syndrome (31.0%; 95% CI, 29.1%-32.9%). Most cardiovascular disease risk factors were independent risk factors for all CAS stages (eg, hypertension: 1.60 [95% CI, 1.60-1.61] for increased cIMT, 1.62 [95% CI, 1.62-1.63] for CP, and 1.48 [95% CI, 1.45-1.51] for CS). Moreover, the magnitude of the association between several cardiovascular disease risk factors and increased cIMT and CP differed between the sexes and geographic regions.

**Conclusions and Relevance:**

These findings suggest that nearly one-quarter of Chinese adults have increased cIMT or CP. The burden of this disease is unevenly distributed across geographic regions and subpopulations and may require different levels of local planning, support, and management. Addressing these disparities is crucial for effectively preventing and managing cardiovascular and cerebrovascular diseases in China.

## Key Points

**Question:**

What is the prevalence of carotid atherosclerosis (CAS) in the general population, in high-risk populations, and in different geographic regions in China?

**Findings:**

This cross-sectional study of more than 10.7 million Chinese adults found prevalences of increased carotid intima-media thickness (26.2%), carotid plaque (21.0%), and carotid stenosis (0.56%). The Chinese population with cardiovascular risk factors, including older age, male sex, residence in northern regions, diabetes, hypertension, dyslipidemia, metabolic syndrome, higher inflammation levels, higher uric acid levels, and higher platelet counts, exhibited a statistically significant higher burden of all subtypes of CAS.

**Meaning:**

The huge burden found in the high-risk subpopulations in this study emphasizes the necessity of identifying and monitoring CAS progression in disease management programs for those with cardiovascular risk factors.

## Teaser

This cross-sectional study of Chinese adults examines the prevalence and associated risk factors of subclinical atherosclerotic carotid disease at the national and provincial levels.

## Introduction

Carotid atherosclerosis (CAS), a main cause of ischemic stroke, can begin early in life and remain latent and asymptomatic for long periods before progressing to advanced stages.^1^ Early-stage phenotypes of CAS, such as increased carotid intima-media thickness (cIMT), and advanced-stage phenotypes, such as carotid plaque (CP) and carotid stenosis (CS), are indicators of stroke.^2,3^ To our knowledge, few studies have investigated the prevalence of various CAS phenotypes in China using nationally representative samples. Therefore, national estimates of CAS prevalence and etiologic studies are of urgent importance for understanding the magnitude of the challenge the country is facing and developing effective strategies for primary prevention, management, and informing stakeholders.

In the absence of a nationally representative study on the prevalence of CAS in China, Song and colleagues^4^ conducted a systematic review and meta-analysis to determine the prevalence of increased cIMT and CP in asymptomatic individuals. Additionally, a previous epidemiologic study also extrapolated CP prevalence using estimates from subregions in China conducted in 2014.^5^ Notably, all previous studies on the prevalence of CAS in China have been based on samples from a limited number of geographically confined communities, which falls far short of representing the entire nation.^5,6^ Moreover, a recent meta-analysis revealed that risk factors for increased cIMT and CP vary significantly across geographic regions.^4^ Given the significant regional variations in lifestyle and economic circumstances, there is a critical need to conduct nationwide studies on CAS burden in different subpopulations with risk factors and in different geographic regions.

To address this knowledge gap, we conducted the first, to our knowledge, national-level cross-sectional study of the general population undergoing routine health checkups in China. Our study aimed to estimate the prevalence of subclinical atherosclerotic carotid disease at the national and provincial levels and in high-risk subpopulations and to investigate the separate and joint effects of the associated risk factors for the disease in this population.

## Methods

### Study Population

Details of the study design, population characteristics, and quality control have been published previously^7^ and are described in eAppendix 1 in Supplement 1. In brief, this is a nationwide, population-based, multicenter, and large-scale cross-sectional study in which participants were recruited from all 31 provincial-level administrative divisions in mainland China. The study was conducted using data from the Meinian Healthcare Group database between January 1, 2017, and June 30, 2022. The Meinian Health Group is the largest health checkup chain in China, providing comprehensive checkup services for the population. All participants who underwent at least 1 checkup, including a carotid artery ultrasound examination, and were 20 years or older at the first checkup made up the study population. For those who attended at least 2 checkups, only the last physical examination record was retained for analyses. A total of 10 772 091 eligible participants were identified. The institutional review board of Peking University approved the study protocol. Because the analyses only used anonymous data, the institutional review board waived individual informed consent.

To ensure the accuracy of our results, we checked for missing information regarding the participants’ age, sex, and geographic region, but no missing data were found. Furthermore, blood samples were collected from all participants (n = 10 772 091). In addition, we excluded participants with a history of cancer (n = 38 116). Ultimately, a total of 10 733 975 participants were included in the analytical sample and were distributed across geographic regions with different socioeconomic development (eFigures 1 and 2 in Supplement 1). All of our procedures followed the Strengthening the Reporting of Observational Studies in Epidemiology (STROBE) reporting guideline.

### Assessment of CAS

Trained sonographers used a Doppler ultrasound system (Mindray DC-8, Mindray North America) equipped with a 3- to 12-MHz high-resolution linear array transducer to conduct carotid ultrasonography. Participants were required to remain in a supine position with their heads turned 45° to the contralateral side of the artery. The measurements were conducted over a minimum of 10-mm length on both sides at the far wall in the common carotid artery, longitudinal and perpendicular to the ultrasound beam, in lateral view, at least 5-mm proximal to the bifurcation in an area with clearly defined lumen-intima, and in a region free of plaque. The cIMT was calculated as the distance from the edge of the first echogenic line to the edge of the second echogenic line. Increased cIMT was defined as 1.0 mm or greater.^8^

The procedure for detecting plaques involved scanning the near and far walls of the common carotid artery, the carotid bifurcation, the external carotid artery, and the internal carotid artery. A plaque was defined according to the latest version of the Mannheim Carotid Intima-Media Thickness and Plaque Consensus: a discrete cIMT of 1.5 mm or larger or focal thickening of 0.5 mm or larger or 50% greater than the surrounding cIMT in any of the aforementioned arterial segments.^9^

The degree of CS was calculated using the diameter measurement method: (normal venous artery diameter − stenosis-residual diameter)/normal venous artery diameter × 00%. Severity of CS was assessed in accordance with the consensus panel gray scale and Doppler ultrasonography criteria; less than 50% was considered mild, 50% to 69% was considered moderate, and 70% or greater was considered severe based on the percentage of stenosis.^10^

### Measures and Definitions of Risk Factors

The study participants were surveyed for demographic information, such as age, sex, and geographic regions, as well as individual medical and medication history, including hypertension, diabetes, dyslipidemia, and tumors. Height, weight, systolic and diastolic blood pressure, and resting heart rate were measured under standardized protocols. All participants provided blood samples following an overnight fast of at least 8 hours. The samples were analyzed using standardized devices and procedures to measure fasting blood glucose, total cholesterol, triglycerides, high-density lipoprotein cholesterol, low-density lipoprotein cholesterol, platelets, uric acid, white blood cells, neutrophils, and lymphocytes. The definition and classification of the aforementioned risk factors are detailed in eAppendix 2 in Supplement 1.

### Statistical Analysis

Our study aims to provide accurate estimates of CAS prevalence (ie, increased cIMT, CP, and CS) according to age, sex, high-risk subpopulations, geographic regions, and economic development levels in the general Chinese population of persons 20 years or older. All prevalence estimates were weighted to represent the total population of Chinese adults (20 years or older) on the basis of Chinese population data from the 2010 census (eAppendix 3 in Supplement 1).

We assessed the normal distribution of continuous variables using the Kolmogorov-Smirnov test. We log-transformed all continuous variables owing to their skewed distributions. Continuous variables are presented as the geometric mean (95% CI) and categorical variables as numbers (percentages). Because of the large sample size, we followed STROBE guidelines and did not use *P* values to assess participant characteristics.^11^ However, we used standardized mean differences to compare differences between the 2 groups. The cutoff values for standardized mean differences were set to 0.2, 0.5, and 0.8, which were interpreted as small, medium, and large differences between the 2 groups, respectively.^12^

For the main analysis, mixed-effect logistic regression models were performed to analyze the association of individual risk factors, the combination of the most influential risk factors, and the interaction of sex and region and risk factors with the prevalence of CAS. Additionally, the dose-response association between individual risk factors and increased cIMT and CP was analyzed using restricted cubic spline logistic regression models. The details of the main analysis and related secondary analyses are given in eTable 1, eFigure 3, and eAppendix 4 in Supplement 1. To ensure the robustness of our findings, we performed several sensitivity analyses (eAppendix 5 in Supplement 1). Because of the potential for type I error due to multiple comparisons, findings for secondary and subgroup analyses should be interpreted as exploratory. SAS software, version 9.4 (SAS Institute Inc) and R software, version 4.2.3 (R Foundation for Statistical Computing) were used for all statistical analyses. A 2-sided *P* < .05 was considered statistically significant.

## Results

### Study Participant Characteristics

From January 1, 2017, to June 30, 2022, this cross-sectional study included 10 733 975 Chinese participants (mean [SD] age, 47.7 [13.4] years; 5 861 566 male [54.6%] and 4 872 409 [45.4%] female; 8 666 501 [80.7%] aged <60 years; 5 939 071 [55.3%] from the North) (eTable 2 in Supplement 1). Risk factors for CAS were highly prevalent among Chinese adults, with a crude prevalence of 17.7% for obesity, 30.8% for hypertension, 8.5% for diabetes, 58.4% for dyslipidemia, and 42.5% for metabolic syndrome (MetS). Table 1 and eTables 3 and 4 in Supplement 1 present the characteristics of the participants overall, as well as according to categories such as age, sex, geographic regions, and various CAS subtypes. In addition, we observed strong and statistically significant correlations among white blood cell count, neutrophil count, lymphocyte counts, and neutrophil-lymphocyte ratio (NLR) (eFigure 4 in Supplement 1).

**Table 1.** Characteristics of the Study Participants According to Sex^a^

Characteristics	Overall (n = 10 733 975)	Males (n = 5 861 566)	Females (n = 4 872 409)	SMD
Age, y	45.8 (45.8-45.8)	45.5 (45.5-45.5)	46.1 (46.1-46.1)	−0.04
Region (north)	5 939 071 (55.3)	3 260 822 (55.6)	2 678 249 (55.0)	0.01
Higher GDP per capita^b^	5 416 324 (50.5)	2 886 920 (49.3)	2 529 404 (51.9)	−0.05
BMI	24.6 (24.6-24.6)	25.4 (25.4-25.4)	23.6 (23.6-23.6)	0.50
FBG, mg/dL	97.8 (97.8-97.8)	100.0 (100.0-100.2)	95.3 (95.3-95.3)	0.23
SBP, mm Hg	126.1 (126.1-126.1)	128.9 (128.9-128.9)	122.9 (122.9-122.9)	0.30
DBP, mm Hg	76.3 (76.3-76.3)	79.2 (79.2-79.2)	72.9 (72.9-72.9)	0.50
Total cholesterol, mg/dL	191.1 (191.1-191.1)	190.7 (190.7-190.7)	191.5 (191.5-191.5)	−0.02
Triglycerides, mg/dL	123.0 (123.0-123.0)	140.7 (140.7-140.7)	104.4 (104.4-104.4)	0.42
LDL-C, mg/dL	108.9 (108.9-108.9)	111.2 (111.2-111.2)	106.2 (106.2-106.2)	0.15
HDL-C, mg/dL	52.1 (52.1-52.1)	49.0 (49.0-49.0)	56.4 (56.4-56.4)	−0.63
Hypertension	3 301 757 (30.8)	2 069 855 (35.3)	1 231 902 (25.3)	0.22
Diabetes	906 772 (8.45)	618 845 (10.6)	287 927 (5.9)	0.23
Dyslipidemia	6 273 214 (58.4)	3 724 404 (63.5)	2 548 810 (52.3)	0.17
MetS, No	4 565 307 (42.5)	2 850 815 (48.6)	1 714 492 (35.2)	0.28
Increased WBCs	197 734 (1.8)	142 215 (2.4)	55 519 (1.1)	0.10
Increased NLR	374 031 (3.5)	220 980 (3.8)	153 051 (3.1)	0.03
Increased heart rate	42 415 (0.4)	21 519 (0.4)	20 896 (0.4)	−0.01
Increased uric acid	2 111 521 (19.7)	1 551 459 (26.5)	560 062 (11.5)	0.39
Increased platelets	224 860 (2.1)	76 222 (1.3)	148 638 (3.0)	−0.12
Increased cIMT	3 394 208 (31.6)	2 095 283 (35.8)	1 298 925 (26.7)	0.20
CP	2 666 296 (24.8)	1 656 821 (28.3)	1 009 475 (20.7)	0.18
CS	61 323 (0.6)	43 571 (0.7)	17 752 (0.4)	0.05
Moderate to severe CS	20 374 (0.2)	16 086 (0.3)	4288 (0.1)	0.04

Abbreviations: BMI, body mass index (calculated as weight in kilograms divided by height in meters squared); cIMT, carotid intima-media thickness; CP, carotid plaque; CS, carotid artery stenosis; DBP, diastolic blood pressure; FBG, fasting blood glucose; GDP, gross domestic product; HDL-C, high-density lipoprotein cholesterol; LDL-C, low-density lipoprotein cholesterol; MetS, metabolic syndrome; NLR, neutrophil-lymphocyte ratio; SBP, systolic blood pressure; SMD, standardized mean difference; WBC, white blood cell.

SI conversion factors: To convert FBG to millimoles per liter, multiply by 0.0555; total cholesterol to millimoles per liter, multiply by 0.0259; triglycerides to millimoles per liter, multiply by 0.0113; and LDL-C and HDL-C to millimoles per liter, multiply by 0.0259.

^a^ Data are presented as geometric least-squares mean (95% CI) or number (percentage) of participants.

^b^ Higher GDP per capita refers to the participant’s city having a GDP value greater than or equal to the median of all cities’ GDP values sorted from smallest to largest.

### Prevalence of CAS

The weighted prevalence of all subtypes of CAS was 26.2% (95% CI, 25.0%-27.4%) for increased cIMT, 21.0% (95% CI, 19.8%-22.2%) for CP, 0.56% (95% CI, 0.36%-0.76%) for CS, and 0.15% (95% CI, 0.13%-0.17%) for moderate to severe CS (Table 2). The prevalence of all CAS grades was higher among older adults (eg, increased cIMT: aged ≥80 years, 92.7%; 95% CI, 92.2%-93.3%), male participants (29.6%; 95% CI, 28.4%-30.7%), those residing in northern China (31.0%; 95% CI, 29.1%-32.9%), and those who had comorbid conditions, such as hypertension (50.8%; 95% CI, 49.7%-51.9%), diabetes (59.0%; 95% CI, 57.8%-60.1%), dyslipidemia (32.1%; 95% CI, 30.8%-33.3%), and MetS (31.0%; 95% CI, 29.1%-32.9%).

**Table 2.** Prevalence of Carotid Atherosclerosis by Study Population Characteristics, 2017-2022

Group	Total participants, No. (%)	Weighted prevalence, % (95% CI)			
		Increased cIMT	CP	CS	Moderate to severe CS
All participants	10 733 975 (100)	26.2 (25.0-27.4)	21.0 (19.8-22.2)	0.6 (0.4-0.8)	0.2 (0.1-0.2)
Participants by year					
2017	335 057 (3.1)	24.1 (22.4-25.8)^a^	18.9 (17.4-20.4)^a^	0.5 (0.3-0.7)^b^	0.2 (0.1-0.2)^a^
2018	1 251 330 (11.7)	23.8 (22.6-25.1)	18.1 (17.1-19.1)	0.6 (0.4-0.7)	0.2 (0.1-0.2)
2019	1 627 270 (15.2)	25.7 (24.3-27.1)	20.5 (19.2-21.9)	0.7 (0.4-1.0)	0.2 (0.2-0.2)
2020	2 136 466 (19.9)	25.2 (23.8-26.7)	20.5 (19.0-21.9)	0.6 (0.3-0.9)	0.1 (0.1-0.2)
2021	3 729 987 (34.8)	26.6 (25.1-28.2)	21.7 (20.1-23.3)	0.5 (0.3-0.7)	0.2 (0.1-0.2)
2022	1 653 865 (15.4)	29.1 (27.6-30.7)	23.5 (21.9-25.1)	0.6 (0.3-0.8)	0.1 (0.1-0.2)
Sex					
Male	5 861 566 (54.6)	29.6 (28.4-30.7)^a^	23.8 (22.6-25.0)^a^	0.7 (0.5-0.8)^c^	0.2 (0.2-0.3)^a^
Female	4 872 409 (45.4)	22.5 (21.3-23.8)	18.1 (16.8-19.3)	0.5 (0.2-0.7)	0.1 (0.1-0.1)
Age group, y					
20-29	951 142 (8.9)	3.4 (2.3-4.5)^a^	2.9 (1.8-4.0)^a^	0.2 (0.0-0.4)^a^	0.0 (0.0-0.0)^a^
30-39	2 399 875 (22.4)	6.7 (5.5-8.0)	5.2 (4.0-6.4)	0.2 (0.0-0.4)	0.0 (0.0-0.0)
40-49	2 426 446 (22.6)	18.9 (17.6-20.1)	13.5 (12.2-14.7)	0.3 (0.1-0.5)	0.0 (0.0-0.0)
50-59	2 889 038 (26.9)	41.2 (39.9-42.4)	30.7 (29.4-32.0)	0.5 (0.3-0.7)	0.1 (0.1-0.1)
60-69	1 489 552 (13.9)	66.8 (65.8-67.9)	54.6 (53.4-55.7)	1.2 (1.0-1.4)	0.4 (0.4-0.5)
70-79	463 975 (4.32)	84.3 (83.6-85.1)	75.2 (74.3-76.1)	2.3 (2.1-2.6)	1.0 (0.9-1.1)
≥80	113 947 (1.1)	92.7 (92.2-93.3)	87.3 (86.7-88.0)	3.9 (3.5-4.3)	1.8 (1.6-2.0)
Region (type I)					
East	2 997 767 (27.9)	25.5 (22.8-28.3)^a^	20.1 (17.2-23.0)^a^	0.4 (0.2-0.5)^a^	0.1 (0.1-0.2)^a^
South	1 115 930 (10.4)	16.0 (14.2-17.8)	11.8 (10.4-13.2)	0.5 (0.0-1.0)	0.1 (0.1-0.1)
Central	1 649 121 (15.4)	28.2 (25.9-30.4)	22.6 (20.5-24.8)	0.6 (0.3-0.8)	0.2 (0.1-0.3)
North	1 672 581 (15.6)	31.5 (27.7-35.3)	25.5 (21.6-29.3)	0.7 (0.3-1.0)	0.3 (0.2-0.3)
Northwest	625 196 (5.8)	30.2 (27.5-32.8)	25.8 (23.4-28.3)	0.3 (0.2-0.4)	0.1 (0.1-0.1)
Southwest	955 946 (8.91)	23.2 (19.8-26.6)	19.3 (16.0-22.7)	0.3 (0.2-0.3)	0.1 (0.1-0.1)
Northeast	1 717 434 (16.0)	28.9 (26.0-31.9)	23.4 (20.6-26.3)	1.0 (0.6-1.3)	0.1 (0.3-0.5)
Region (type II)					
South	4 794 904 (44.7)	20.9 (19.6-22.2)^a^	16.3 (15.0-17.6)^a^	0.4 (0.2-0.6)^c^	0.1 (0.1-0.1)^a^
North	5 939 071 (55.3)	31.0 (29.1-32.9)	25.2 (23.3-27.2)	0.6 (0.5-0.8)	0.3 (0.2-0.3)
BMI					
<18.5	260 478 (2.4)	11.8 (10.5-13.0)^a^	10.0 (8.8-11.2)^a^	0.5 (0.2-0.7)^b^	0.1 (0.1-0.1)^a^
18.5-23.9	4 163 679 (38.8)	20.7 (19.5-21.9)	16.8 (15.6-18.0)	0.5 (0.3-0.7)	0.1 (0.1-0.1)
24.0-27.9	4 415 541 (41.2)	32.0 (30.8-33.2)	25.6 (24.4-26.9)	0.6 (0.5-0.8)	0.2 (0.2-0.2)
≥28.0	1 894 277 (17.7)	31.1 (29.8-32.3)	24.4 (23.1-25.6)	0.5 (0.4-0.7)	0.2 (0.1-0.2)
Diabetes					
Yes	906 772 (8.4)	59.0 (57.8-60.1)^a^	49.8 (48.6-51.0)^a^	1.5 (1.2-1.7)^a^	0.6 (0.5-0.6)^a^
No	9 827 203 (91.6)	23.8 (22.7-25.0)	19.0 (17.8-20.2)	0.5 (0.3-0.7)	0.1 (0.1-0.1)
Hypertension					
Yes	3 301 757 (30.8)	50.8 (49.7-51.9)^a^	42.2 (41.0-43.4)^a^	1.1 (0.9-1.4)^a^	0.4 (0.4-0.5)^a^
No	7 432 218 (69.2)	17.8 (16.6-19.0)	13.8 (12.6-15.1)	0.4 (0.2-0.6)	0.1 (0.1-0.1)
Dyslipidemia					
Yes	6 273 214 (58.4)	32.1 (30.8-33.3)^a^	25.6 (24.3-26.9)^a^	0.6 (0.4-0.8)^a^	0.2 (0.2-0.2)^a^
No	4 460 761 (41.6)	19.4 (18.2-20.5)	15.7 (14.6-16.9)	0.5 (0.3-0.7)	0.1 (0.1-0.1)
MetS					
Yes	4 565 307 (42.5)	40.6 (39.3-41.8)^a^	33.1 (31.9-34.3)^a^	0.9 (0.7-1.0)^a^	0.3 (0.3-0.3)^a^
No	6 168 668 (57.5)	17.8 (16.6-19.0)	14.0 (12.8-15.2)	0.4 (0.2-0.6)	0.1 (0.1-0.1)

Abbreviations: BMI, body mass index (calculated as weight in kilograms divided by height in meters squared); cIMT, carotid intima-media thickness; CP, carotid plaque; CS, carotid artery stenosis; MetS, metabolic syndrome.

^a^ *P* < .001 for difference (*P* for difference can be expressed as whether there is a statistically significant difference in prevalence between subgroups).

^b^ *P* > .05 for difference.

^c^ *P* > .001 and <.05 for difference.

**Figure 1.**
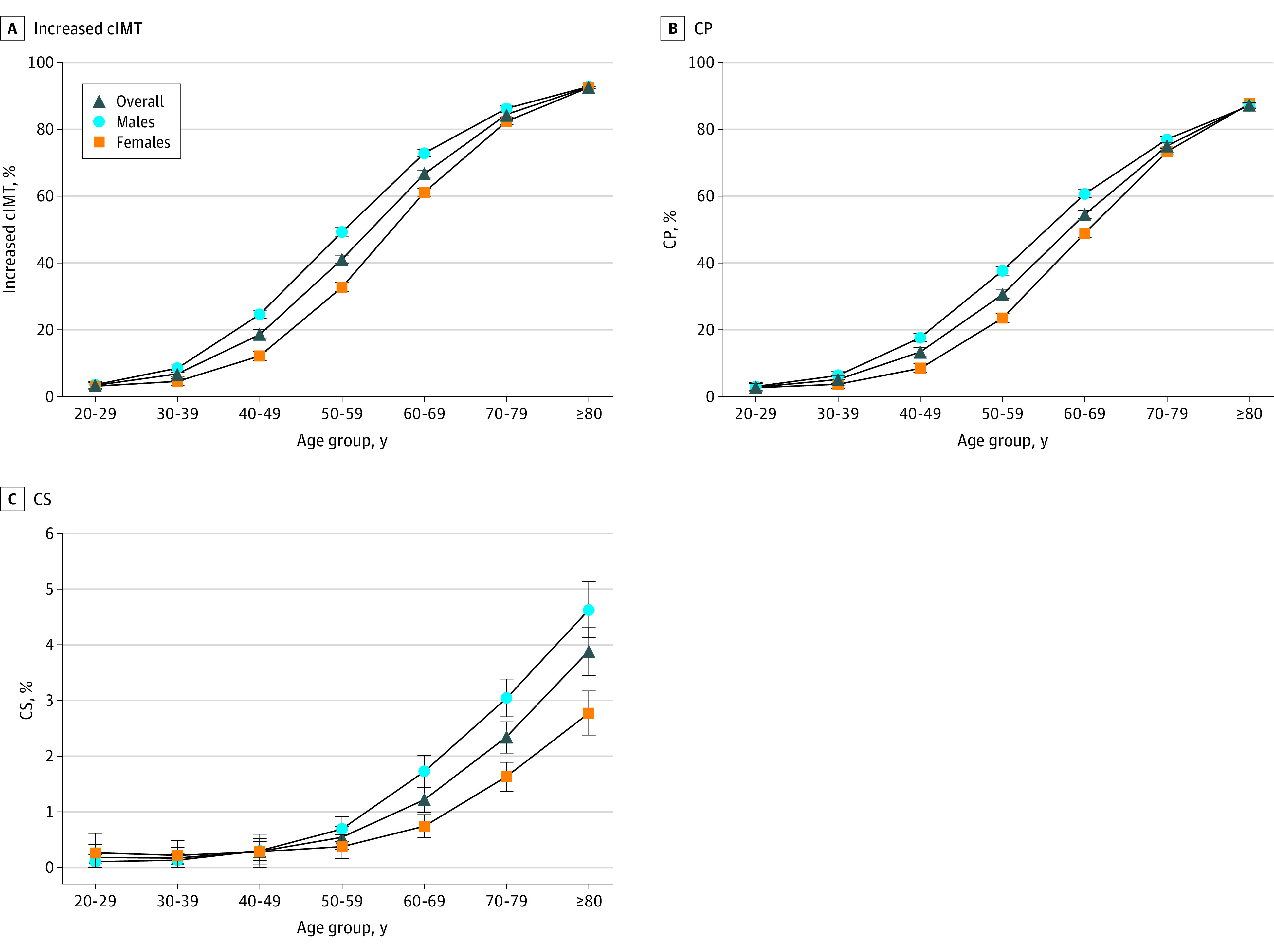
Age- and Sex-Specific Prevalence of Increased Carotid Intima-Media Thickness (cIMT), Carotid Plaque (CP), and Carotid Stenosis (CS) Standardized by Regions Whiskers indicate 95% CIs.

The overall weighted prevalence of increased cIMT, CP, and CS in different age groups was higher in male participants than in female participants (Figure 1). Furthermore, the prevalence of increased cIMT, CP, and CS was negatively associated with gross domestic product per capita (eFigure 5 in Supplement 1). With increasing of the BMI, systolic blood pressure, and low-density lipoprotein cholesterol levels, the prevalence of increased cIMT, CP, and CS also increased, but this finding was not statistically significant (eFigures 6 and 7 in Supplement 1).

**Figure 2.**
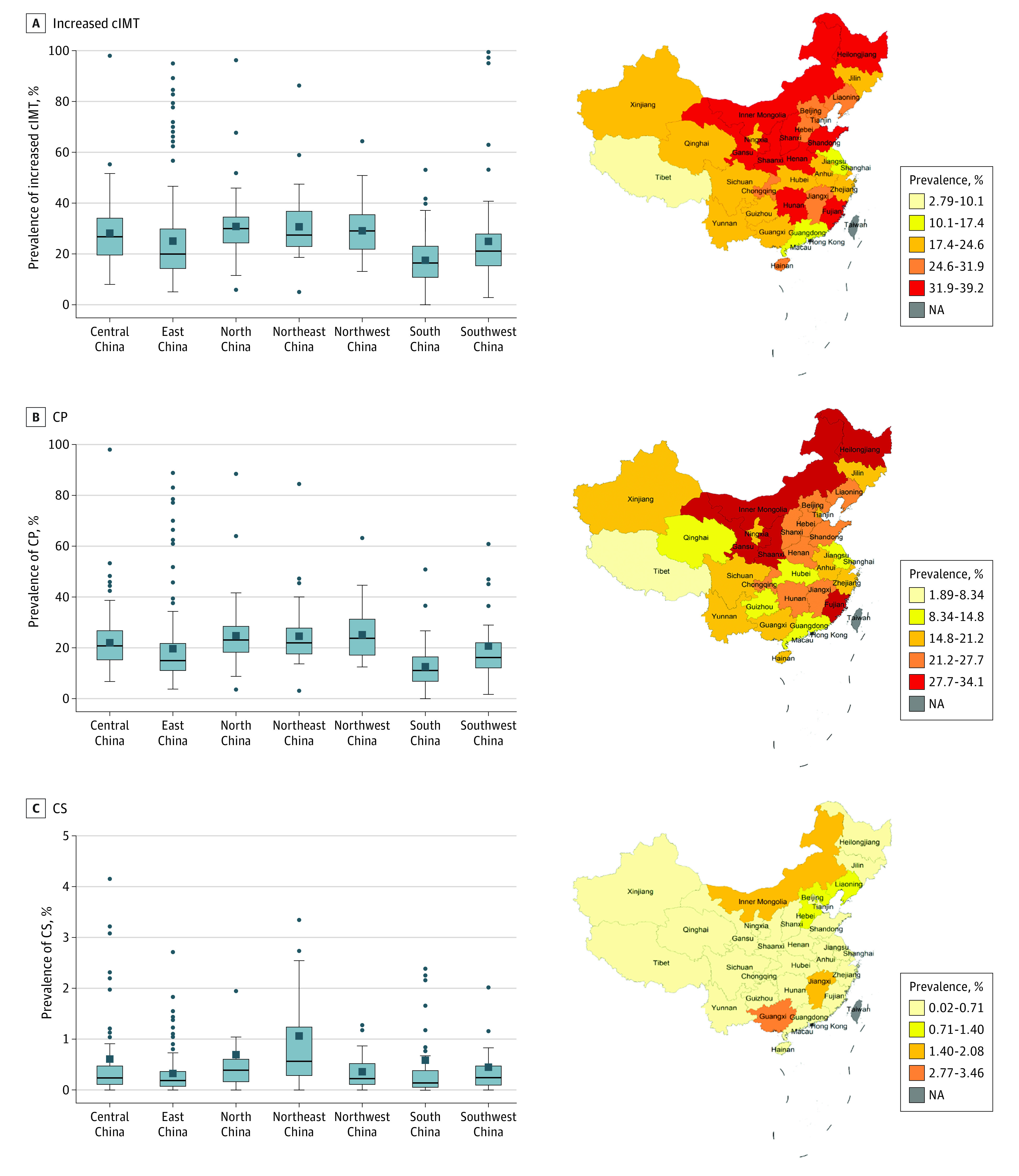
Region-Level Weighted Prevalence of Carotid Atherosclerosis in China Age- and sex-standardized prevalence of carotid atherosclerosis was calculated for each province according to the 2010 National Population Census. The box plots were drawn using the data of the prevalence at center level and grouped according to 7 geographic regions of China. In the box plots, the box edges indicate upper and lower quartiles; middle lines indicate the median; whiskers indicate maximum and minimum values; squares indicate the mean; and small dots indicate represent outliers. cIMT indicates carotid intima-media thickness; CP, carotid plaque; CS, carotid stenosis; NA, not applicable.

Figure 2 illustrates a map of China, with colors representing the prevalence of increased cIMT, CP, and CS per province. The population distribution of patients with CAS (increased cIMT, CP, and CS) was concentrated in the Shandong Province in the East and the Liaoning Province in the Northeast (eFigure 8 in Supplement 1). eFigure 9 in Supplement 1 shows the temporal variation in the weighted prevalence of increased cIMT, CP, and CS in the southern and northern regions since 2017. Detailed results of weighted prevalence are given in eAppendix 3 in Supplement 1.

### Multivariable Analyses of CAS Risk Factors

Regarding distal factors, we observed significant associations between increased cIMT, CP, and CS prevalence and study factors, including age, sex, and geographic region (Table 3). Older age, male sex, and residence in the northern region were independently associated with a higher prevalence of various CAS subtypes after mutual adjustment. In term of intermediate and proximal factors, the multivariable mixed-effects model showed that hypertension and diabetes had the strongest association with increased cIMT, CP, and CS prevalence, followed by MetS, increased white blood cell count, BMI, dyslipidemia, increased heart rate, increased uric acid levels, increased platelet count, and increased NLR. Notably, among participants with hypertension, the odds ratios (ORs) were 1.60 (95% CI, 1.60-1.61) for increased cIMT, 1.62 (95% CI, 1.62-1.63) for CP, and 1.48 (95% CI, 1.45-1.51) for CS compared with those without hypertension. Stratified analyses suggested that sex and geographic region might play important roles as modifiers of these associations (eFigures 10 and 11 in Supplement 1). The sensitivity analysis results are described in detail in eAppendix 3 and eTables 5 to 10 in Supplement 1.

**Table 3.** Association Between the Risk Factors and Carotid Atherosclerosis

Characteristic	OR (95% CI)		
	Increased cIMT^a^	CP	CS
Distal factors^b^			
Age group, y			
20-29	0.03 (0.03-0.03)^c^	0.04 (0.04-0.04)^c^	0.16 (0.15-0.18)^c^
30-39	0.07 (0.07-0.07)	0.08 (0.08-0.08)	0.18 (0.17-0.19)
40-49	0.27 (0.27-0.27)	0.28 (0.28-0.29)	0.37 (0.36-0.38)
50-59	1.00 [Reference]	1.00 [Reference]	1.00 [Reference]
≥60	4.25 (4.23-4.27)	4.05 (4.04-4.07)	3.82 (3.74-3.89)
Sex			
Female	1.00 [Reference]	1.00 [Reference]	1.00 [Reference]
Male	2.14 (2.13-2.15)	1.99 (1.98-1.99)	2.19 (2.15-2.23)
Region			
South	1.00 [Reference]	1.00 [Reference]	1.00 [Reference]
North	1.67 (1.52-1.85)	1.80 (1.63-1.98)	1.76 (1.47-2.10)
Intermediate factors^d^			
BMI			
<18.5	0.99 (0.97-1.00)^c^	1.05 (1.03-1.06)^c^	1.33 (1.25-1.42)^c^
18.5-23.9	1.00 [Reference]	1.00 [Reference]	1.00 [Reference]
24.0-27.9	1.15 (1.15-1.16)	1.10 (1.10-1.10)	0.92 (0.90-0.93)
≥28.0	1.28 (1.27-1.29)	1.17 (1.16-1.17)	0.82 (0.81-0.85)
Obesity	1.18 (1.17-1.18)	1.10 (1.09-1.10)	0.87 (0.85-0.89)
Increased WBCs	1.33 (1.31-1.34)	1.34 (1.32-1.36)	1.37 (1.30-1.44)
Increased NLR	1.07 (1.06-1.08)	1.09 (1.08-1.10)	1.20 (1.15-1.24)
Increased heart rate	1.15 (1.12-1.18)	1.24 (1.20-1.27)	1.00 (0.89-1.13)
Increased uric acid	1.12 (1.11-1.12)	1.14 (1.14-1.15)	1.10 (1.08-1.12)
Increased platelets	1.09 (1.08-1.11)	1.10 (1.09-1.12)	1.28 (1.20-1.36)
Proximal factors			
Hypertension^e^			
No	1.00 [Reference]	1.00 [Reference]	1.00 [Reference]
Yes	1.60 (1.60-1.61)	1.62 (1.62-1.63)	1.48 (1.45-1.51)
Diabetes^e^			
No	1.00 [Reference]	1.00 [Reference]	1.00 [Reference]
Yes	1.62 (1.61-1.62)	1.62 (1.61-1.63)	1.51 (1.48-1.54)
Dyslipidemia^e^			
No	1.00 [Reference]	1.00 [Reference]	1.00 [Reference]
Yes	1.26 (1.26-1.27)	1.22 (1.21-1.22)	1.05 (1.03-1.07)
MetS^f^			
No	1.00 [Reference]	1.00 [Reference]	1.00 [Reference]
Yes	1.48 (1.47-1.48)	1.50 (1.49-1.50)	1.40 (1.37-1.42)

Abbreviations: BMI, body mass index (calculated as weight in kilograms divided by height in meters squared); cIMT, carotid intima-media thickness; CP, carotid plaque; CS, carotid artery stenosis; MetS, metabolic syndrome; NLR, neutrophil to lymphocyte ratio; OR, odds ratio; WBC, white blood cell.

^a^ Obtained by using multivariable mixed-effect logistic regression analysis.

^b^ Adjusted for age, sex, gross domestic product per capita, and region for distal factors.

^c^ *P* < .001 for trend.

^d^ Adjusted for age, sex, gross domestic product per capita, region, BMI, WBC count, NLR, heart rate, uric acid level, and platelet count for intermediate factors.

^e^ Additionally adjusted for hypertension, diabetes, and dyslipidemia for proximal factors.

^f^ Adjusted for age, sex, gross domestic product per capita, region, BMI, WBC count, NLR, heart rate, uric acid level, and platelet count for MetS.

### Cumulative Effect and Dose-Response Manner of Multiple Risk Factors

Using mutually adjusted analyses, we calculated ORs for the risk of increased cIMT and CP with multiple simultaneous risk factors (eFigure 12 in Supplement 1). In participants 60 years or older, those with hypertension, those with diabetes, male participants, and those from the North, the ORs increased from 4.25 (95% CI, 4.23-4.27) to 14.51 (95% CI, 14.21-14.82) for increased cIMT and from 4.05 (95% CI, 4.04-4.07) to 10.79 (95% CI, 10.61-10.97) for CP (eAppendix 4 in Supplement 1). The dose-response analysis conducted in this study revealed a nonlinear association between increased cIMT and CP with continuous variables such as BMI, white blood cell count, NLR, and uric acid concentrations (eFigures 13 and 14 and eAppendix 4 in Supplement 1).

## Discussion

This study used national checkup data from 2017 to 2022 to examine the distribution of all subtypes of CAS in China and identify cross-sectional factors associated with its prevalence. Our research reveals 3 principal findings. First, the study reported the nationwide prevalence of CAS, including increased cIMT, CP, and CS using, for the first time that we are aware of, consistent methods and a representative sample with coverage of all provinces in China. The prevalence of all subtypes of CAS varied substantially across age, sex, and province, with higher prevalence among older individuals, male participants, and those living in northern regions. Second, we identified several key clinical, demographic, and socioeconomic factors associated with all subtypes of CAS. These factors comprised age, sex, gross domestic product per capita, geographic region, BMI, white blood cell count, NLR, heart rate, uric acid concentration, platelet count, hypertension, diabetes, dyslipidemia, and MetS. The combined effect of these major factors substantially increased the population-level risk of increased cIMT and CP. Third, broad heterogeneity in the factors associated with increased cIMT and CP across sexes and regions of China was observed. This finding highlights the importance of sex- and region-specific analyses when analyzing increased cIMT and CP risk factors.

A recent systematic review comprising 59 studies revealed a broad range in the global prevalence of CAS among individuals aged 30 to 79 years in 2020. Globally, the prevalence of increased cIMT ranged from 16.9% to 41.3%, the prevalence of CP ranged from 13.2% to 31.5%, and the prevalence of CS ranged from 1.1% to 2.1%.^13^ In 2018, a meta-analysis estimated the pooled prevalence of increased cIMT and CP in China to be 27.2% and 20.2%, respectively.^4^ The prevalence of increased cIMT and CP reported in this study aligns with these previous findings. Specifically, the weighted prevalence of increased cIMT and CP in the checkup population was 26.2% and 21.0%, respectively. Compared with the prevalence of CP reported in the China Kadoorie Biobank (CKB) study, a large cross-sectional investigation covering multiple geographic regions in China, our estimate of CP was relatively low (21.0% vs 30.9%).^5^ This difference may be attributed to our study’s relatively younger age structure (mean age, 48 years) compared with that in the CKB study (mean age, 59 years) because the risk of developing CP tends to increase with advancing age. The weighted prevalence of CS in our study (0.56%) was lower than that reported in most prior studies,^6,14^ primarily because of the relatively healthy checkup participants included in our study. Our analysis further identified a marked predominance of all subtypes of CAS in male participants and in northern populations, consistent with the meta-analysis conducted by Song et al^4,13^ and previous studies.^15^ The prevalence of increased cIMT and CP has been found to increase annually, irrespective of sex or geographic region (eFigure 9 in Supplement 1). Furthermore, in conjunction with previous research,^13^ our study demonstrated a noteworthy increase in the prevalence of increased cIMT and CP among individuals aged 50 years or older (Figure 1). Considering demographic aging in China and decreasing birth rates leading to a shrinking overall population,^16^ it is expected that the incidence of increased cIMT and CP is expected to persistently increase, placing a substantial burden on the Chinese health care system. This finding highlights the significance of targeted interventions for high-risk populations, particularly older adults. By prioritizing prevention and early detection strategies within this age group, it may be possible to alleviate the adverse effects of CAS and reduce the overall burden of cardiovascular and cerebrovascular diseases in China. Future studies should investigate the underlying mechanisms responsible for age-related variations in the prevalence of CAS, thereby providing a more comprehensive understanding of the condition and its implications for public health. To date, there are no nationally representative estimates of the various subtypes of CAS burden derived from the checkup population in China. Nonetheless, previous studies have reported comparable associations between various subtypes of CAS and sociodemographic characteristics along with other risk factors, such as hypertension.

Individuals with various subtypes of CAS are at a higher risk of developing cardiovascular diseases, particularly CP, which has been indicated as a crucial factor in assessing cardiovascular health.^17^ Therefore, it is imperative to identify the risk factors associated with various subtypes of CAS and implement targeted interventions to prevent disease progression. We conducted a study and found that the factors associated with all subtypes of CAS were consistent with those reported in previous studies conducted in China and other countries. For instance, a meta-analysis indicated that male sex, diabetes, and hypertension were common risk factors for increased cIMT and CP.^13^ Meta-analyses and prospective studies have reported that serum uric acid levels are significantly associated with increased cIMT and CP.^18,19^ Both cross-sectional and cohort studies have reported a significant association between inflammatory cytokines (ie, white blood cell count and NLR), resting heart rate, platelet count, and MetS and the risk of increased cIMT and CP.^20,21,22,23,24,25,26,27,28^ Additionally, a cross-sectional study conducted among young adults in China revealed that multiple factors, including age, BMI, triglyceride levels, and low-density lipoprotein cholesterol levels were positively associated with increased cIMT and CP.^29^ Two additional cross-sectional studies of Asian populations reported significant associations among age, BMI, hypertension, diabetes, dyslipidemia, and CS.^30,31^ Furthermore, BMI presented a J-shaped dose-response association with increased cIMT and CP in our study, consistent with previous findings regarding the association between BMI and stroke.^32^ On the basis of previous research^32^ and the results of this study, it is speculated that maintaining body weight within the normal range has the strongest protective effect on cardiovascular and cerebrovascular diseases. In this study, in addition to sociodemographic factors, hypertension and diabetes were found to be most closely associated with increased cIMT, CP, and CS, which agrees with previous epidemiologic investigations.^13,30,33^ Nearly half of the patients with hypertension and diabetes in the current study had increased cIMT and CP. Moreover, there was a significant association among hypertension, diabetes, and the prevalence of increased cIMT and CP in both male and female participants, as well as among individuals from the northern and southern regions. The early detection and management of hypertension and diabetes may help slow the progression of atherosclerotic complications. Importantly, our findings indicate that increased cIMT and CP was significantly associated with multiple coexisting risk factors. As the number of risk factors increased, such as old age, hypertension, diabetes, male sex, and residence in northern regions, the risk of developing increased cIMT and CP gradually increased. This finding highlights the importance of screening, diagnosing, and preventing CAS by specifically targeting vulnerable subpopulations, such as male individuals, older individuals, residents of northern regions, and populations with unfavorable cardiovascular risk factors.

To the best of our knowledge, this is the first study to report the prevalence of CAS (defined as increased cIMT, CP, and CS) in different provinces and geographic regions of China. The distribution of CAS prevalence exhibits geographic variations in a north-to-south gradient across China, with the highest burden observed in the northern and central regions, consistent with a nationally representative study of stroke burden.^34^ Geographic variations in the CAS burden may be related to differences in the prevalence of risk factors for CAS across these regions. Our study identified hypertension, diabetes, and obesity as risk factors for CAS, with the highest prevalence of these risk factors reported in the northern region compared with other regions.^35,36,37^ Additionally, geographic variations in the CAS burden might also be partly attributable to location-related lifestyles and genetic backgrounds.^36,38^ Lower socioeconomic status and poor access to health care services may also contribute to geographic disparities in the CAS burden. Geographic variation in CAS implies that specific regions should be prioritized in allocating medical resources and should receive more attention in the diagnosis, screening, and prevention of the disease. This prioritization is crucial for reducing the geographic disparities in CAS and cardiovascular diseases.

### Limitations

Our study has some limitations. First, information regarding education, smoking status, alcohol consumption, physical activity, and diet was not available during the participants’ checkups. This limited our ability to explore the potential risk factors associated with CAS, which resulted in residual confounding factors. However, we adjusted for the province-level average years of education, smoking prevalence, and drinking prevalence as covariates in the model to reduce the effect of residual confounding factors on the association of risk factors with CAS. Second, probability sampling is often used in descriptive studies, providing scientific evidence for extrapolating study results to the population, and is considered the gold standard for ensuring external validity. However, the population included in this study comprised individuals who underwent health checkups and who may have unique characteristics compared with the general population. Therefore, this study lacks representativeness of the entire population and might have resulted in an underestimation of the prevalence in China. Nevertheless, we accounted for the age and sex structure of the population in the prevalence estimates to enable external comparisons. Third, this study estimated the prevalence of CAS based on participants enrolled from 2017 to 2022. A longer interval increases the likelihood of measurement bias, which can either underestimate or overestimate the prevalence of CAS. Fourth, owing to the cross-sectional nature of this study, the observed associations may have been influenced by reverse causation, particularly for lifestyle-related factors. However, we excluded patients with cancer to reduce the possible effects of lifestyle changes on potential reverse causality.

## Conclusions

Our study highlights the significant burden of CAS in China, with increased cIMT and CP being highly prevalent in high-risk subpopulations. Notably, the prevalence rates of all CAS stages (defined as increased cIMT, CP, and CS) were significantly higher among older individuals, male individuals, individuals residing in northern regions, and those with diabetes, hypertension, dyslipidemia, MetS, higher inflammation levels, higher uric acid levels, and higher platelet counts. These findings provide valuable evidence for implementing health policies and identifying high-risk populations in China to prevent the development of cardiovascular and cerebrovascular diseases in both the general population and high-risk subgroups.

## Notes

Supplement 1. **eAppendix 1.** The Quality Control Process of Meinian Health Check-Up **eAppendix 2.** Measures and Definitions of Risk Factors **eAppendix 3.** Description of the Calculation Method and Results of Weighted Prevalence **eAppendix 4.** Description of Main Analysis and Related Secondary Analyses **eAppendix 5.** Description of the Methods and Results From Sensitivity Analysis **eTable 1.** The Proportions of Missing Values of Each Variable, and the Distribution of Values of Each Variable Before and After Imputation **eTable 2.** General Characteristics of the Study Population, 2017-2022 (n = 10 733 975) **eTable 3.** Characteristics of Participants According to Classification of Age and Region (n = 10 733 975) **eTable 4.** Characteristics of Participants According to CAS Status (n = 10 733 975) **eTable 5.** Association Between the Risk Factors as Continuous Variables and CAS (Adjusted ORs and 95% Confidence Intervals; n = 10 733 975) **eTable 6.** Association Between the Risk Factors and CAS Using Multivariable Logistic Regression Model (Adjusted ORs and 95% Confidence Intervals; n = 10 733 975) **eTable 7.** Prevalence of CAS by the Characteristics of Study Population Using Information From the First Check-Ups, 2017-2022 (n = 9 847 132) **eTable 8.** Association Between the Risk Factors and CAS Using Complete Data (Adjusted ORs and 95% Confidence Intervals; n = 7 292 261) **eTable 9.** Association Between the Risk Factors and CAS Using Full Model (Adjusted ORs and 95% Confidence Intervals; n = 10 733 975) **eTable 10.** Association Between the Risk Factors and CAS (Adjusted ORs and 95% Confidence Intervals; n = 10 733 975) **eFigure 1.** Flowchart of the Study Participation **eFigure 2.** Geographical Distribution of the Check-Up Centers Included Into the Study **eFigure 3.** Conceptual Model Risk Factors for CAS **eFigure 4.** Correlations Between Participants’ Blood Relevant Parameters **eFigure 5.** Association Between Prevalence of Different Grades of CAS, and GDP Per Capita **eFigure 6.** Prevalence of Increased cIMT, CP and CS by BMI, SBP, and LDL-C Quintiles After Age, Gender, and Regional Standardization **eFigure 7.** Association Between Prevalence of Different Grades of CAS and BMI, SBP, and LDL-C Levels **eFigure 8.** Relative Contribution to the Total Burden of CAS by Region **eFigure 9.** Joinpoint Regression Analysis of the Trend for the Prevalence of CAS in the South and North Populations From 2017 to 2022 **eFigure 10.** Association Between the Risk Factors and Increased cIMT, Stratified by Sex and Region **eFigure 11.** Association Between the Risk Factors and CP, Stratified by Sex and Region **eFigure 12.** Additive Risk Plot Showing Odds Ratios for Increased cIMT and CP When Considering Multiple Risk Factors Simultaneously **eFigure 13.** The Restricted Cubic Spline for the Association Between Risk Factors (A-E) and Increased cIMT in the Overall Population **eFigure 14.** The Restricted Cubic Spline for the Association Between Risk Factors (A-E) and CP in the Overall Population

Supplement 2.
Data Sharing Statement
