# Supplementary material for: National and Provincial-Level Prevalence and Risk Factors of Carotid Atherosclerosis in Chinese Adults
Source: JAMA Netw Open. 2024 Jan 11;7(1):e2351225. doi: 10.1001/jamanetworkopen.2023.51225 (PMC10784858; doi:10.1001/jamanetworkopen.2023.51225)
Supplement: Supplement 1. — eAppendix 1. The Quality Control Process of Meinian Health Check-Up eAppendix 2. Measures and Definitions of Risk Factors eAppendix 3. Description of the Calculation Method and Results of Weighted Prevalence eAppendix 4. Description of Main Analysis and Related Secondary Analyses eAppendix 5. Description of the Methods and Results From Sensitivity Analysis eTable 1. The Proportions of Missing Values of Each Variable, and the Distribution of Values of Each Variable Before and After Imputation eTable 2. General Characteristics of the Study Population, 2017-2022 (n = 10 733 975) eTable 3. Characteristics of Participants According to Classification of Age and Region (n = 10 733 975) eTable 4. Characteristics of Participants According to CAS Status (n = 10 733 975) eTable 5. Association Between the Risk Factors as Continuous Variables and CAS (Adjusted ORs and 95% Confidence Intervals; n = 10 733 975) eTable 6. Association Between the Risk Factors and CAS Using Multivariable Logistic Regression Model (Adjusted ORs and 95% Confidence Intervals; n = 10 733 975) eTable 7. Prevalence of CAS by the Characteristics of Study Population Using Information From the First Check-Ups, 2017-2022 (n = 9 847 132) eTable 8. Association Between the Risk Factors and CAS Using Complete Data (Adjusted ORs and 95% Confidence Intervals; n = 7 292 261) eTable 9. Association Between the Risk Factors and CAS Using Full Model (Adjusted ORs and 95% Confidence Intervals; n = 10 733 975) eTable 10. Association Between the Risk Factors and CAS (Adjusted ORs and 95% Confidence Intervals; n = 10 733 975) eFigure 1. Flowchart of the Study Participation eFigure 2. Geographical Distribution of the Check-Up Centers Included Into the Study eFigure 3. Conceptual Model Risk Factors for CAS eFigure 4. Correlations Between Participants’ Blood Relevant Parameters eFigure 5. Association Between Prevalence of Different Grades of CAS, and GDP Per Capita eFigure 6. Prevalence of Increased cIMT, CP and CS by BMI, SBP, [file jamanetwopen-e2351225-s001.pdf]

Supplementary Online Content

Fu J, Deng Y, Ma Y, et al. National and provincial-level prevalence and risk factors of carotid atherosclerosis in Chinese adults, 2017-2022. JAMA Netw Open. 2024;7(1):e2351225. doi:10.1001/jamanetworkopen.2023.51225

eAppendix 1. The Quality Control Process of Meinian Health Check-Up
eAppendix 2. Measures and Definitions of Risk Factors
eAppendix 3. Description of the Calculation Method and Results of Weighted Prevalence
eAppendix 4. Description of Main Analysis and Related Secondary Analyses
eAppendix 5. Description of the Methods and Results From Sensitivity Analysis
eTable 1. The Proportions of Missing Values of Each Variable, and the Distribution of Values of Each Variable Before and After Imputation
eTable 2. General Characteristics of the Study Population, 2017-2022 (n=10,733,975)
eTable 3. Characteristics of Participants According to Classification of Age and Region (n=10,733,975)
eTable 4. Characteristics of Participants According to CAS Status (n=10,733,975)
eTable 5. Association Between the Risk Factors as Continuous Variables and CAS (Adjusted ORs and 95% Confidence Intervals; n=10,733,975)
eTable 6. Association Between the Risk Factors and CAS Using Multivariable Logistic Regression Model (Adjusted ORs and 95% Confidence Intervals; n=10,733,975)
eTable 7. Prevalence of CAS by the Characteristics of Study Population Using Information From the First Check-Ups, 2017-2022 (n=9,847,132)
eTable 8. Association Between the Risk Factors and CAS Using Complete Data (Adjusted ORs and 95% Confidence Intervals; n=7,292,261)
eTable 9. Association Between the Risk Factors and CAS Using Full Model (Adjusted ORs and 95% Confidence Intervals; n=10,733,975)
eTable 10. Association Between the Risk Factors and CAS (Adjusted ORs and 95% Confidence Intervals; n=10,733,975)
eFigure 1. Flowchart of the Study Participation
eFigure 2. Geographical Distribution of the Check-Up Centers Included Into the Study
eFigure 3. Conceptual Model Risk Factors for CAS
eFigure 4. Correlations Between Participants' Blood Relevant Parameters

© 2024 Fu J et al. *JAMA Network Open.*

eFigure 5. Association Between Prevalence of Different Grades of CAS, and GDP Per Capita
eFigure 6. Prevalence of Increased cIMT, CP and CS by BMI, SBP, and LDL-C Quintiles After Age, Gender, and Regional Standardization
eFigure 7. Association Between Prevalence of Different Grades of CAS and BMI, SBP, and LDL-C Levels
eFigure 8. Relative Contribution to the Total Burden of CAS by Region
eFigure 9. Joinpoint Regression Analysis of the Trend for the Prevalence of CAS in the South and North Populations From 2017 to 2022
eFigure 10. Association Between the Risk Factors and Increased cIMT, Stratified by Sex and Region
eFigure 11. Association Between the Risk Factors and CP, Stratified by Sex and Region
eFigure 12. Additive Risk Plot Showing Odds Ratios for Increased cIMT and CP When Considering Multiple Risk Factors Simultaneously
eFigure 13. The Restricted Cubic Spline for the Association Between Risk Factors (A-E) and Increased cIMT in the Overall Population
eFigure 14. The Restricted Cubic Spline for the Association Between Risk Factors (A-E) and CP in the Overall Population

This supplementary material has been provided by the authors to give readers additional information about their work.

eAppendix 1. The quality control process of Meinian health check-up

Routine health check-ups have emerged as an essential healthcare service in China, playing a crucial role in early disease detection and prevention at the population level. Over the past decade, health check-ups have gained widespread popularity, with an estimated 30% of the population undergoing such examinations in 2020. In China, providers of health check-up services offer diverse packages encompassing a range of tests, including physical examinations, laboratory analyses, and imaging procedures. The Chinese government actively promotes the provision of health check-ups by encouraging employers to offer them as part of employee welfare programs. Notably, around 80% of individuals visiting health check-up centers are sponsored by their employers, with individual participation typically being cost-free. Consequently, the choice of examinations is predominantly influenced by employers rather than being based on personal preferences or medical recommendations. As a result, variations in economic status and health conditions among individuals do not significantly impact the types of examinations received.

As the largest health check-up chain in China, Meinian Healthcare Group offers a wide range of health check-up services catering to the adult population. Its network of check-up centers spans across all 31 provinces in mainland China. The majority of Meinian's health check-up participants comprise employees from government organizations, public and private enterprises, as well as members of social organizations. Additionally, freelancers and retirees from nearby communities also take part in these check-ups. At Meinian check-up centers, each participant undergoes a comprehensive examination that includes physical assessments, laboratory tests, and imaging scans. To ensure accurate results, all participants are required to fast for a minimum of eight hours prior to the check-up. Trained doctors and technicians conduct all tests and examinations within the centers.

The check-up process at Meinian Healthcare Group underwent rigorous quality control measures. Firstly, standardized training on the check-up process and equipment operation procedures was provided to all doctors, technicians, and nurses at the centers. Secondly, biological samples were promptly transferred to central laboratories in each province for testing. These laboratories participated in External Quality Assessment overseen by the National Center for Clinical Laboratories of the National Health and Family Planning Commission, ensuring

© 2024 Fu J et al. *JAMA Network Open.*

that test results met established quality standards. Additionally, Meinian Healthcare Group's Department of Quality Control conducted monthly internal assessments to uphold and enhance laboratory quality. Thirdly, the establishment and implementation of a standardized operating procedure for the check-up process across the entire system aimed to standardize procedures and quality control measures at every center. The quality control team conducted unannounced inspections, encompassing on-site checks of operational procedures, documentation reviews, and customer inquiries, thereby providing comprehensive oversight of the check-up process. Fourthly, all check-up data was entered into a web-based data management system at the center level. Data managers at the Meinian Healthcare Group's Department of Medical Information Center in Shanghai and data statisticians at the Meinian Institute of Health in Beijing monitored data flow and quality using validation procedures and progress reports for all centers. Any data inconsistencies were promptly identified and rectified by engaging with the respective centers. These robust quality control measures ensured the reliable and accurate collection of check-up process data.

© 2024 Fu J et al. *JAMA Network Open.*

eAppendix 2. Measures and definitions of risk factors

The study participants were surveyed for demographic information, such as age, sex, and geographical regions, as well as individual medical and medication history, including hypertension, diabetes, dyslipidemia, and tumors. Additionally, cardiovascular disease risk factors were assessed, which included measurements of height, weight, systolic and diastolic blood pressure (SBP/DBP), and resting heart rate (HR), using a standard protocol. The geographical regions were categorized into Type I (East, South, Central, North, Northwest, Southwest, and Northeast) and Type II (South and North). Body mass index (BMI) was calculated by dividing weight in kilograms by height in meters squared, and then categorized into four groups: < 18.5 (underweight), 18.5-23.9 (normal), 24.0-27.9 (overweight), and ≥ 28.0 (obesity). [1] Hypertension was defined as SBP/DBP ≥ 140/90 mmHg, confirmed hypertension medication use, or self-reported history of hypertension. Increased HR was identified if HR > 100 times/min. City-level GDP per capita was categorized using the median as a cutoff to represent socioeconomic status.

In this study, to account for the socioeconomic and lifestyle impact on CAS, multivariate models made additional adjustments for province-level average educational years, smoking prevalence, and drinking prevalence in sensitivity analysis. These covariates were divided into three levels according to their tertiles and then analyzed as categorical variables in the model. Details of these covariates are presented as follows. Data on province-level average educational years and city-level GDP per capita were collected from the China Statistical Yearbook for 2020. Educational years were categorized into low (< 9.75 years), middle (9.75 years ~ 10.10 years), and high (≥ 10.11 years). GDP per capita was also categorized into low (< 60,611 CNY), middle (60,611 CNY ~ 92,175 CNY), and high (≥ 92,176 CNY). Province-level smoking prevalence was extracted from the results of Wang et al. [2], which was estimated based on a national cross-sectional survey in 2013. Smoking prevalence was categorized into below average (< 24.3%), average (24.3% ~ 25.0%), and above average (> 25.1%). Province-level drinking prevalence was obtained from the 2002 Chinese National Nutrition and Health Survey (2002 NNHS), which was a stratified multigrade cluster sampling study covering 132 sample sites from all 31 provinces. [3] Drinking prevalence was then categorized into low, middle, and high according to the rank of the prevalence in males and females.

© 2024 Fu J et al. *JAMA Network Open.*

All participants provided blood samples following an overnight fast of at least 8 hours. The samples were analyzed using standardized devices and procedures to measure fasting blood glucose (FBG), total cholesterol (TC), triglyceride (TG), high density lipid-cholesterol (HDL-C), low density lipid-cholesterol (LDL-C), platelet count (PLT), uric acid (UA), white blood cells (WBC), neutrophil, and lymphocyte. Form these measurements, we calculated the neutrophil-to-lymphocyte ratio (NLR) as the absolute neutrophil count divided by the absolute lymphocyte count. Participants were classified as having diabetes if they had a FBG ≥ 7.0 mmol/L, were using diabetes medication, or had a history of diabetes. [4] Dyslipidemia was defined as having TC of ≥ 5.17 mmol/L, TG of ≥ 1.7 mmol/L, LDL-C of ≥ 3.37 mmol/L, HDL-C < 1.00 mmol/L or using dyslipidemia medication. [5] Metabolic syndrome (MetS) was defined based on the 2009 harmonized criteria of MetS. [6] Participants were considered to have MetS if they presented two or more of the following components: 1) elevated BP (SBP ≥ 130 mm Hg and/or diastolic BP ≥ 85 mm Hg) or self-reported hypertension or taking antihypertensive medication, 2) elevated TG (≥ 1.7 mmol/L), or using lipid-lowering medication, 3) reduced HDL (< 1.0 mmol/L in males; < 1.3 mmol/L in females) or using lipid-lowering medication, 4) elevated fasting glucose (≥ 5.56 mmol/L) or self-reported diabetes or using antidiabetic drugs. Increased PLT was identified if PLT > 350.0×10^9/L. Increased UA was identified if UA > 420.0 μmol/L for males or UA > 357.0 μmol/L for females. [7] Increased WBC was identified if WBC > 10.0×10^9/L. Increased neutrophil was identified if neutrophil > 6.30×10^9/L. Increased lymphocyte was identified if lymphocyte > 3.20×10^9/L. Increased NLR was identified if NLR > 3.53. [8]

References

1. Zhou BF. Effect of body mass index on all-cause mortality and incidence of cardiovascular diseases--report for meta-analysis of prospective studies open optimal cut-off points of body mass index in Chinese adults. Biomed Environ Sci. 2002;15(3):245-252.
2. Wang M, Luo X, Xu S, et al. Trends in smoking prevalence and implication for chronic diseases in China: serial national cross-sectional surveys from 2003 to 2013. Lancet Respir Med. 2019;7(1):35-45.

© 2024 Fu J et al. *JAMA Network Open.*

3. L. W. Summary report of China Nutrition and Health Survey 2002. Beijing: People's Medical Publishing House. 2005.
4. American Diabetes A. Diagnosis and classification of diabetes mellitus. Diabetes Care. 2014;37 Suppl 1:S81-90.
5. Expert Panel on Detection E, Treatment of High Blood Cholesterol in A. Executive Summary of The Third Report of The National Cholesterol Education Program (NCEP) Expert Panel on Detection, Evaluation, And Treatment of High Blood Cholesterol In Adults (Adult Treatment Panel III). JAMA. 2001;285(19):2486-2497.
6. Alberti KG, Eckel RH, Grundy SM, et al. Harmonizing the metabolic syndrome: a joint interim statement of the International Diabetes Federation Task Force on Epidemiology and Prevention; National Heart, Lung, and Blood Institute; American Heart Association; World Heart Federation; International Atherosclerosis Society; and International Association for the Study of Obesity. Circulation. 2009;120(16):1640-1645.
7. Branch of Cardiovascular Physicians CMDA. [Consensus of Chinese experts on diagnosis and treatment of asymptomatic hyperuricemia combined with cardiovascular diseases]. CHINA MODERN MEDICINE. 2009;16(24):4-8.
8. Forget P, Khalifa C, Defour JP, Latinne D, Van Pel MC, De Kock M. What is the normal value of the neutrophil-to-lymphocyte ratio? BMC Res Notes. 2017;10(1):12.

© 2024 Fu J et al. *JAMA Network Open.*

eAppendix 3. Description of the calculation method and results of weighted prevalence

Age, sex, and geographical regions were specified as important risk factors of interest *a priori*. To better represent the epidemiology of CAS (i.e., increased cIMT, CP, and CS) in Chinese adults, we assigned each participant a weight coefficient based on the weight for the unequal distribution of samples across provinces and the poststratification weight, which harmonized the age and sex structure of the study population with that of the 2010 census of the Chinses population. The weighted prevalence and the corresponding 95% confidence intervals (CIs) were estimated using the Taylor series linearization method incorporating the cluster effect of centers with stratification by province. The Taylor series linearization method estimates variance from the variance among cluster in the sample and combines stratum variance estimates to compute the overall variance estimates. All prevalence reported in this study are weighted. The weighted prevalence between subgroups was compared using the Rao-Scott adjusted χ^2 test. It improves the performance in the problem of goodness-of-fit and can help to avoid misleading results generated by clustering in the sample. Additionally, we analyzed the weighted CAS prevalence stratified by age and sex. To gain insights into province-level patterns in weighted prevalence and its relative contribution to the total burden of CAS, we constructed heatmaps of China. We also used the Joinpoint regression program to analyze sex- and region-specific trends in the weighted prevalence of CAS in Chinese adults between 2017 to 2022. We calculated the average annual percentage change (AAPC) for the entire period from 2017 to 2022, and the estimated annual percent change (APC) was calculated for each segment. Moreover, we also conducted an ecological analysis to explore the association between the prevalence of CAS and GDP per capita.

Figure 2 illustrates a map of China, with colors representing the prevalence of increased cIMT, CP, and CS per province. The weighted prevalence of increased cIMT, CP and CS was highest in the northern Inner Mongolia Autonomous Region, Shaanxi Province, and Guangxi Zhuang Autonomous Region, respectively, at 39.2% (95% CI, 30.6%-47.9%), 34.1% (95% CI, 29.4%-38.9%) and 3.46% (95% CI, 0.00%-7.29%) (**Figure 2**; data not shown). Inner Mongolia Autonomous Region showed the second-highest prevalence of CP and CS, at 32.3% (95% CI, 23.9%-40.8%) and 1.46% (95%CI, 0.00%-3.30%), respectively. Conversely, several provinces

from the southwest, south, and east regions had the lowest prevalence, including Tibet, Jiangsu, Guangdong, and Guizhou (e.g., Guizhou for increased cIMT: 17.8%; 95% CI, 11.9%-23.7%; CP: 14.7%; 95% CI, 9.29%-20.0%; CS: 0.29%; 95% CI, 0.11%-0.47%). The population distribution of patients with CAS (increased cIMT, CP and CS) was concentrated in Shandong Province in the east and Liaoning Province in the northeast (**eFigure 8**). In combination, these two provinces contributed almost 25% of cases of various CAS subtypes, followed by nearly 9.0% of cases in Henan and 7.0% in Hebei.

eFigure 9 shows the temporal variation in the weighted prevalence of increased cIMT, CP and CS in the southern and northern regions since 2017. From 2017 to 2022, the prevalence of increased cIMT (APC/AAPC:3.67, $P = .001$) and CP (APC/AAPC:5.24, $P < .001$) increased significantly in both the South and North over time, with no statistically significant difference in this trend between the North and South ($P > 0.05$). The trend of both sexes showed a significant increase over the period from 2017 to 2022 in both the North (APC/AAPC: 3.27, $P < .001$) and South (APC/AAPC: 4.29, $P < .001$) for increased cIMT, with no statistically significant differences between females and males in this trend ($P > .05$). Meanwhile, for CP, the trend for both sexes showed a significant increase from 2017 to 2022 in the South (APC/AAPC:5.47, $P < .001$), whereas in the North, the trend for both sexes exhibited a declining pattern that was not significant (APC:2017-2018:-10.7, $P = .37$), but increased significantly thereafter (APC:2018-2022:6.80, $P < .001$; AAPC:3.0, $P = .26$). There were also no statistically significant differences in the prevalence trends between males and females in either the South or the North ($P > .05$). In terms of CS, from 2017 to 2022, the trend for both sexes showed a significant decrease in the South (APC/AAPC: -6.27, $P = .016$), but not in the North and nationally.

© 2024 Fu J et al. *JAMA Network Open.*

eAppendix 4. Description of methods and results of main and related secondary analyses

For the main analysis, we considered the status of CAS as a dependent variable and sociodemographic and health-related variables as independent variables. We selected risk factors based on published literatures on CAS and cardiovascular disease-related risk factors, including age, sex, geographical regions, BMI, hypertension, diabetes, dyslipidemia, MetS, WBC, NLR, HR, PLT, and UA. Since the participants in the same physical examination center may not be independent from each other due to the shared environment and facilities, violating the independence assumption of regression models, we applied a mixed-effect model with study factors at the participant level as a fixed-effect term, and center as a random-effect term. We used a conceptual framework [1] to categorize the study factors for CAS into distal factors (e.g., age, sex, geographical regions, and GDP per capita), intermediate factors (e.g., BMI, WBC, NLR, HR, UA, and PLT) and proximal factors (e.g., hypertension, diabetes, dyslipidemia, and MetS), assuming the former influenced the latter. This framework determined the factors to retain in the multivariable models. We treated the distal factors as potential confounders of the association between intermediate factors and the prevalence of CAS **(eFigure 3)**. Similarly, the distal and intermediate factors were considered as potential confounders for the association between proximal factors and CAS. Linear trends were tested for significance by including the categorical variables as ordinal variables in the model. For simplicity, only associations of risk factors with increased cIMT and CP are reported in the following results. To investigate whether sex and region modified the association of risk factors with increased cIMT and CP, we used the χ^2 likelihood ratio test to assess interactions on multiplicative scale. Stratified analysis was further performed to assess the associations of risk factors with increased cIMT and CP in participants of different sexes and living in different geographic locations. We selected the most influential risk factors based on statistical significance and coefficient magnitude to generate an additive risk model for the cumulative probability of increased cIMT and CP when considering multiple categorical variables simultaneously. Furthermore, we estimated the dose-response association between risk factors and the prevalence of increased cIMT and CP using restricted cubic spline logistic regression, where extreme variables values ≤ 0.01st percentile and ≥ 99.9th percentile were excluded to avoid the influence of extreme values. We detected

© 2024 Fu J et al. *JAMA Network Open.*

multicollinearity among covariates in the final model using variance inflation factors (VIFs), with VIFs exceeding 10 indicating multicollinearity. All VIFs ranged from 1.05 to 1.59, indicating no collinearity issue. The proportion of missing values in the study participants was 21.9% for HR, and less than 20% for other variables **(eTable 1)**. Most missing values were generated due to inconsistencies in physical examination items. For continuous missing value, we performed multiple imputation (with five imputations) using the chained equations method.[2]

Using mutually adjusted analyses, we calculated ORs for the risk of increased IMT and CP with multiple simultaneous risk factors **(eFigure 12)**. The reference levels were defined as previously described. Participants aged 60 years or older had ORs of 4.250 (95%CI, 4.233-4.268) and 4.052 (95%CI, 4.036-4.068) for developing increased cIMT and CP, respectively, compared to those aged 50 to 59 years. If these participants also had hypertension, the OR increased to 9.863 (95%CI, 9.815-9.910) and 8.445 (95%CI, 8.407-8.482). Similarly, if they had diabetes, the OR increased to 10.487 (95%CI, 10.375-10.600) and 8.798 (95%CI, 8.716-8.881). Furthermore, if these participants were males and had the aforementioned risk factors, the OR increased to 13.218 (95%CI, 13.018-13.421) and 10.357 (95%CI, 10.225-10.490). Finally, if they were from the North and had all other factors, the OR for increased cIMT and CP increased to 14.509 (95%CI, 14.206-14.819) and 10.791 (95%CI, 10.610-10.974).

The dose-response analysis conducted in this study revealed a non-linear association between increased cIMT and CP with continuous variables such as BMI, WBC count, NLR, and UA concentrations (*P* for overall association < .001 and *P* for non-linear < .001; **eFigures 13 and 14**). Additionally, there was a non-linear dose-response association between PLT and increased cIMT (*P* for overall association < .001 and *P* for non-linear < .001), whereas the association between PLT and CP was linear (*P* for overall association < .001 and *P* for non-linear = .14). Among these variables, BMI and WBC demonstrated a J-shaped relationship with increased cIMT and CP. Our analysis indicated that the threshold concentration of BMI and WBC were 21.0 kg/m^2 and 4.50×10^9/L for increased cIMT and 22.0 kg/m^2 and 4.50×10^9/L for CP, respectively. Above the threshold, we observed significant associations between increased cIMT and CP prevalence with BMI and WBC. The adjusted ORs for log-transformed in BMI and WBC were 3.279 (95%CI: 3.228, 3.331) and 2.107 (95%CI: 2.088, 2.126) for increased cIMT. For CP, the adjusted ORs were 2.275 (95%CI: 2.238, 2.313) and 2.124 (95%CI: 2.104,

© 2024 Fu J et al. *JAMA Network Open.*

2.144), respectively.

References

1. Price AJ, Crampin AC, Amberbir A, et al. Prevalence of obesity, hypertension, and diabetes, and cascade of care in sub-Saharan Africa: a cross-sectional, population-based study in rural and urban Malawi. Lancet Diabetes Endocrinol. 2018;6(3):208-222.

2. Azur MJ, Stuart EA, Frangakis C, Leaf PJ. Multiple imputation by chained equations: what is it and how does it work? Int J Methods Psychiatr Res. 2011;20(1):40-49.

eAppendix 5. Description of the methods and results from sensitivity analysis

In order to ensure the robustness of our findings, we performed several sensitivity analyses. Firstly, we adjusted for log-transformed age, BMI, WBC, PLT, UA, HR, and NLR as continuous variables instead of categorical variables. Secondly, we conducted multivariable logistic regression analysis to re-examine the association between risk factors and CAS. Thirdly, we only included data from the first physical examination of participants who underwent multiple physical examinations in order to analyze the prevalence of CAS in the general population and across subgroups. Fourthly, to check whether the imputation of missing values affected the results, we repeated the mixed-effect logistic regression analyses among participants with complete data on the risk factors (n=7,292,261). Fifth, all risk factors were included in the mixed-effect model for mutual adjustment, and the association between risk factors and CAS was observed. Finally, due to the lack of lifestyle-related variables in this study, to adjust the impact of the above variables, we included province-level average educational years, smoking prevalence, and drinking prevalence as covariates into the multivariable model to assess their impact. [1,2]

Several sensitivity analyses were performed in this study to ensure the robustness of our results. Firstly, we transformed categorical variables into continuous variables and found that our main results remained unchanged (e**Table 4**). Secondly, we conducted a multivariable logistic regression analysis to investigate the association between risk factors and CAS. our main results remained unaffected (e**Table 5**). Thirdly, when we retained only the first physical examination record for analyses, the results of our sensitivity analysis for estimating CAS prevalence were slightly lower but broadly consistent (e**Table 6**). Fourthly, when repeating the analyses among participants without any missing values on risk factors, the results were similar (e**Table 7**). Fifthly, when we adjusted for all risk factors in a mixed-effect model, the results remained unchanged except that the association between HR and CAS became weaker (e**Table 8**). Lastly, we further explored the associations between risk factors and CAS by including regional average educational years, smoking prevalence, and drinking prevalence in the model (**eTable 9**). The results showed similar associations between risk factors and CAS.

References

© 2024 Fu J et al. *JAMA Network Open.*

1. Wang M, Luo X, Xu S, et al. Trends in smoking prevalence and implication for chronic diseases in China: serial national cross-sectional surveys from 2003 to 2013. Lancet Respir Med 2019;7(1):35-45. doi: 10.1016/S2213-2600(18)30432-6 [published Online First: 2018/11/30]
2. L. W. Summary report of China Nutrition and Health Survey 2002. Beijing: People's Medical Publishing House. 2005

© 2024 Fu J et al. *JAMA Network Open.*

eTable 1. The proportions of missing values of each variable, and the distribution of values of each variable before and after imputation

Variables	Missing, N (%)	Before imputation		After imputation	
		Mean (SD)	Median (P_{25}, P_{75})	Mean (SD)	Median (P_{25}, P_{75})
**BMI (kg/m^2)**	843,653 (7.86)	24.8 (3.62)	24.6 (22.3, 27.0)	24.8 (3.54)	24.5 (22.2, 26.9)
**SBP (mmHg)**	666,410 (6.21)	127.3 (19.4)	125.0 (113.0, 139.0)	127.5 (19.1)	123 (112, 136)
**DBP (mmHg)**	666,581 (6.21)	77.3 (12.4)	76.0 (68.0, 85.0)	77.4 (12.2)	75 (67, 84)
**TC (mmol/L)**	229,093 (2.13)	5.05 (1.02)	4.97 (4.35, 5.66)	5.05 (1.00)	4.89 (4.28, 5.55)
**TG mmol/L**	231,449 (2.16)	1.69 (1.40)	1.33 (0.91, 2.00)	1.69 (1.39)	1.34 (0.92, 2.00)
**HDL-C, mmol/L**	1,242,490 (11.6)	1.39 (0.37)	1.35 (1.15, 1.57)	1.39 (0.32)	1.35 (1.16, 1.57)
**LDL-C, mmol/L**	683,633 (6.37)	2.94 (0.83)	2.88 (2.36, 3.44)	2.94 (0.82)	2.88 (2.37, 3.43)
**FBG, mmol/L**	556,113 (5.18)	5.55 (1.48)	5.25 (4.84, 5.76)	5.56 (1.43)	5.27 (4.85, 5.79)
**WBC (10^9/L)**	562,701 (5.24)	6.05 (5.73)	5.81 (4.91, 6.90)	6.02 (1.69)	5.81 (4.94, 6.86)

© 2024 Fu J et al. *JAMA Network Open.*

Variables	Missing, N (%)	Before imputation		After imputation	
		Mean (SD)	Median (P_{25}, P_{75})	Mean (SD)	Median (P_{25}, P_{75})
**Neutrophil (10^9/L)**	544,233 (5.07)	3.56 (3.70)	3.36 (2.71, 4.15)	3.54 (1.28)	3.36 (2.73, 4.13)
**Lymphocyte (10^9/L)**	515,878 (4.81)	2.02 (2.05)	1.92 (1.57, 2.32)	2.01 (1.39)	1.91 (1.58, 2.30)
**HR (times/min)**	2,353,996 (21.9)	71.3 (9.37)	71.0 (65.0, 76.0)	71.4 (8.52)	71.1 (66.0, 76.0)
**Uric acid (μmol/L)**	374,580 (3.49)	333.9 (95.6)	324.0 (263.7, 394.0)	333.5 (94.6)	324.0 (264.0, 393.0)
**Platelet (10^9/L)**	324,361 (3.02)	222.5 (57.9)	218.0 (184.0, 257.0)	222.2 (56.8)	218.0 (184.0, 256.0)

BMI body mass index, DBP diastolic blood pressure, FBG fasting blood glucose, GDP gross domestic product, HDL-C high-density lipoprotein cholesterol, HR heart rate, LDL-C low-density lipoprotein cholesterol, MetS Metabolic syndrome, NLR neutrophil to lymphocyte ratio, P percentile, SBP systolic blood pressure, TC total cholesterol, TG triglycerides, WBC white blood cells

© 2024 Fu J et al. *JAMA Network Open.*

eTable 2. General characteristics of the study population, 2017-2022 (n=10,733,975)

Characteristics	Classifications	Median (IQR)/n (%)
**Age (years)**	20-29	951,142 (8.86) [a]
	30-39	2,399,875 (22.4)
	40-49	2,426,446 (22.6)
	50-59	2,889,038 (26.9)
	60-~	2,067,474 (19.3)
**Sex (%)**	Males	5,861,566 (54.6)
	Females	4,872,409 (45.4)
**Region (type I) (%)**	East	2,997,767 (27.9)
	South	1,115,930 (10.4)
	Central	1,649,121 (15.4)
	North	1,672,581 (15.6)
	Northwest	625,196 (5.82)
	Southwest	955,946 (8.91)
	Northeast	1,717,434 (16.0)
**Region (type II) (%)**	South	4,794,904 (44.7)
	North	5,939,071 (55.3)
**GDP per capita (%, RMB)**	< Median	5,317,651 (49.5)
	≥ Median	5,416,324 (50.5)
**COVID-19 pandemic (%)**	Yes	7,520,318 (70.1)
	No	3,213,657 (29.9)
**BMI (kg/m^2)**	< 18.5	260,478 (2.43)
	18.5-23.9	4,163,679 (38.8)
	24.0-27.9	4,415,541 (41.2)
	≥ 28.0	1,894,277 (17.7)
**FBG (mmol/L)**	Continuous	5.27 (4.85, 5.79) [b]
**SBP (mmHg)**	Continuous	126.0 (114.0, 139.0)
**DBP (mmHg)**	Continuous	77.0 (69.0, 85.0)
**TC (mmol/L)**	Continuous	4.98 (4.36, 5.65)
**TG (mmol/L)**	Continuous	1.34 (0.92, 2.00)
**LDL-C (mmol/L)**	Continuous	2.88 (2.37, 3.43)
**HDL-C (mmol/L)**	Continuous	1.35 (1.16, 1.57)
**Hypertension (%)**	Yes	3,301,757 (30.8)
	No	7,432,218 (69.2)
**Diabetes (%)**	Yes	906,772 (8.45)
	No	9,827,203 (91.6)
**Dyslipidemia (%)**	Yes	6,273,214 (58.4)
	No	4,460,761 (41.6)
**MetS (%)**	Yes	4,565,307 (42.5)
	No	6,168,668 (57.5)
**Characteristics**	**Classifications**	**Median (IQR)/n (%)**
**WBC (10^9/L)**	> 10.0	197,734 (1.84)
	≤ 10.0	1,0536,241 (98.2)
**Neutrophil (10^9/L)**	> 6.30	284,244 (2.65)
	≤ 6.30	10,449,731 (97.4)
**Lymphocyte (10^9/L)**	> 3.20	368,218 (3.43)
	≤ 3.20	10,365,757 (96.6)
**NLR**	> 3.53	374,031 (3.48)
	≤ 3.53	10,359,944 (96.5)
**HR (times/min)**	> 100	42,415 (0.40)
	≤ 100	10,691,560 (99.6)
	> 428 for males; > 357 for females	2,111,521 (19.7)

© 2024 Fu J et al. *JAMA Network Open.*

**Uric acid (μmol/L)**	≤ 428 for males; ≤ 357 for females	8,622,454 (80.3)
**Platelet (10^9/L)**	> 350	224,860 (2.09)
	≤ 350	10,509,115 (97.9)
**Increased cIMT (%)**	Yes	3,394,208 (31.6)
	No	7,339,767 (68.4)
**CP (%)**	Yes	2,666,296 (24.8)
	No	8,067,679 (75.2)
**CS (%)**	Yes	61,323 (0.57)
	No	10,672,652 (99.4)
**Moderate to severe CS (%)**	Yes	20,374 (0.19)
	No	10,713,601 (99.8)

BMI body mass index, cIMT carotid intima-media thickness, CP carotid plaque, CS carotid artery stenosis, DBP diastolic blood pressure, FBG fasting blood glucose, GDP gross domestic product, HDL-C high-density lipoprotein cholesterol, HR heart rate, LDL-C low-density lipoprotein cholesterol, MetS Metabolic syndrome, NLR neutrophil to lymphocyte ratio, SBP systolic blood pressure, TC total cholesterol, TG triglycerides, WBC white blood cells.

[a] Categorical variables were expressed as n (%) (all such values).

[b] Continuous variables were expressed as median (IQR).

© 2024 Fu J et al. *JAMA Network Open.*

eTable 3. Characteristics of participants according to classification of age and region (n=10,733,975)

Characteristics	Mean (95%CI) Group of age G1	Group of age G5	SMD	Region South	Region North	SMD
**Subjects (n)**	951,142	2,067,474		4,794,904	5,939,071	
**Age (years)**	26.1 (26.1, 26.1) [a]	66.9 (66.9, 66.9)	8.76	44.8 (44.8, 44.8)	46.5 (46.5, 46.6)	0.13
**Sex (males, No. (%))**	523,391 (55.0) [b]	1,082,465 (52.4)	-0.05	2,600,744 (54.2)	3,260,822 (54.9)	0.01
**Region (North, No. (%))**	471,335 (49.6)	1,260,985 (61.0)	0.23	-	-	-
**GDP per capita (≥ Median, No. (%))**	544,367 (57.2)	988,238 (47.8)	-0.19	3,232,195 (67.4)	2,184,129 (36.8)	-0.64
**BMI (kg/m2)**	23.1 (23.1, 23.1)	24.9 (24.9, 24.9)	0.44	24.0 (24.0, 24.0)	25.1 (25.1, 25.1)	0.31
**FBG (mmol/L)**	4.93 (4.93, 4.93)	5.84 (5.84, 5.84)	0.80	5.28 (5.28, 5.28)	5.56 (5.56, 5.56)	0.21
**SBP (mmHg)**	116.4 (116.4, 116.4)	139.4 (139.4, 139.5)	1.40	123.3 (123.3, 123.3)	128.5 (128.5, 128.5)	0.28
**DBP (mmHg)**	70.5 (70.5, 70.5)	78.6 (78.6, 78.6)	0.79	74.6 (74.5, 74.6)	77.8 (77.8, 77.8)	0.27
**TC (mmol/L)**	4.53 (4.53, 4.53)	5.09 (5.09, 5.09)	0.60	4.98 (4.98, 4.98)	4.93 (4.93, 4.93)	-0.05
**TG (mmol/L)**	1.08 (1.08, 1.08)	1.42 (1.42, 1.42)	0.31	1.37 (1.37, 1.37)	1.40 (1.40, 1.40)	0.01
**LDL-C (mmol/L)**	2.53 (2.53, 2.53)	2.90 (2.90, 2.90)	0.48	2.85 (2.85, 2.85)	2.80 (2.80, 2.80)	-0.05
**HDL-C (mmol/L)**	1.35 (1.35, 1.35)	1.38 (1.38, 1.38)	0.10	1.37 (1.37, 1.37)	1.34 (1.34, 1.34)	-0.08
**Hypertension, No. (%)**	75,369 (7.92)	1,190,053 (57.6)	1.25	1,250,649 (26.1)	2,051,108 (34.5)	0.18
**Diabetes, No. (%)**	7,214 (0.76)	349,346 (16.9)	0.60	329,582 (6.87)	577,190 (9.72)	0.10
**Dyslipidemia, No. (%)**	341,517 (35.9)	1,338,557 (64.7)	0.59	2,807,876 (58.6)	3,465,338 (58.4)	-0.004
**MetS, No. (%)**	167,010 (17.6)	1,264,481 (61.2)	1.00	1,779,704 (37.1)	2,785,603 (46.9)	0.20
**Increased WBC, No. (%)**	21,453 (2.26)	31,799 (1.54)	-0.05	83,213 (1.74)	114,521 (1.93)	0.01
**Increased NLR, No. (%)**	25,016 (2.63)	96824 (4.68)	0.11	170,121 (3.55)	203,910 (3.43)	-0.01
**Increased HR (%)**	5,927 (0.62)	9,534 (0.46)	-0.02	17,854 (0.37)	24,561 (0.41)	0.01
**Increased uric acid (%)**	245,889 (25.9)	349,486 (16.9)	-0.22	1,090,359 (22.7)	1,021,162 (17.2)	-0.14
**Increased platelet (%)**	25,307 (2.66)	24630 (1.19)	-0.11	87,249 (1.82)	137,611 (2.32)	0.03
**Characteristics**	**Mean (95%CI) Group of age G1**	**Group of age G5**	**SMD**	**Group of age South**	**North**	**SMD**
**Increased cIMT, No. (%)**	30,992 (3.26)	1,518,324 (73.4)	2.09	1,215,770 (25.4)	2,178,438 (36.7)	0.25
**CP, No. (%)**	27,367 (2.88)	1,280,773 (62.0)	1.63	928,077 (19.4)	1,738,219 (29.3)	0.23
**CS, No. (%)**	753 (0.08)	38,294 (1.85)	0.18	20,322 (0.42)	41,001 (0.69)	0.04
**Moderate to severe CS, No. (%)**	12 (0.00)	16,153 (0.78)	0.13	4,326 (0.090)	16,048 (0.27)	0.04

© 2024 Fu J et al. *JAMA Network Open.*

Abbreviations: G1 and G5: We divided the age into five groups, from the lowest group to the highest group, they were respectively 20-29 y (G1), 30-39 y, 40-49 y, 50-59 y, and ≥ 60 y (G5). BMI body mass index, cIMT carotid intima-media thickness, CP carotid plaque, CS carotid artery stenosis, DBP diastolic blood pressure, FBG fasting blood glucose, G group, GDP gross domestic product, HDL-C high-density lipoprotein cholesterol, HR heart rate, LDL-C low-density lipoprotein cholesterol, MetS Metabolic syndrome, NLR neutrophil to lymphocyte ratio, SBP systolic blood pressure, SMD standardized mean difference, TC total cholesterol, TG triglycerides, WBC white blood cells.

[a] Geometric least square mean (95% confidence interval) (all such values).

[b] Categorical variables are expressed as number (percentage) of participants.

© 2024 Fu J et al. *JAMA Network Open.*

eTable 4. Characteristics of participants according to CAS status (n=10,733,975)

Characteristics	Mean (95%CI)								
	Increased cIMT		SMD	CP		SMD	CS		SMD
	Yes	No		Yes	No		Yes	No	
Subjects (n)	3,394,208	7, 339,767	-	2,666,296	8,067,679	-	61,323	10,672,652	-
**Age (years)**	57.1 (57.1, 57.1) [a]	41.3 (41.3, 41.3)	1.37	57.8 (57.8, 57.8)	42.4 (42.4, 42.4)	1.29	61.3 (61.2, 61.5)	45.7 (45.7, 45.7)	1.17
**Sex (males, No. (%))**	2,095,283 (61.7) [b]	3,766,283 (51.3)	0.21	1, 656,821 (62.1)	4,204,745 (52.1)	0.20	43,571 (71.1)	5, 817,995 (54.5)	0.35
**Region (North, No. (%))**	2,178,438 (64.2)	3,760,633 (51.2)	0.26	1,738,219 (65.2)	4,200,852 (52.1)	0.27	41,001 (66.9)	5,898,070 (55.3)	0.24
**GDP per capita (≥ Median, No. (%))**	1,563,989 (46.1)	3, 852,335 (52.5)	-0.13	1, 210,964 (45.4)	4, 205,360 (52.1)	-0.13	22,623 (36.9)	5,393,701 (50.5)	-0.28
**BMI (kg/m²)**	25.2 (25.2, 25.2)	24.3 (24.3, 24.3)	0.24	25.2 (25.2, 25.2)	24.3 (24.3, 24.3)	0.20	24.8 (24.8, 24.9)	24.6 (24.6, 24.6)	0.07
**FBG (mmol/L)**	5.81 (5.80, 5.81)	5.27 (5.27, 5.27)	0.41	5.84 (5.84, 5.84)	5.30 (5.30, 5.31)	0.40	6.02 (6.01, 6.03)	5.43 (5.43, 5.43)	0.40
**SBP (mmHg)**	135.3 (135.2, 135.3)	122.1 (122.1, 122.1)	0.72	136.0 (135.9, 136.0)	123.0 (123.0, 123.1)	0.70	139.5 (139.3, 139.6)	126.1 (126.1, 126.1)	0.67
**DBP (mmHg)**	79.8 (79.8, 79.8)	74.8 (74.8, 74.8)	0.41	79.7 (79.7, 79.8)	75.2 (75.2, 75.2)	0.37	79.1 (79.0,79.2)	76.3 (76.3, 76.3)	0.25
**TC (mmol/L)**	5.13 (5.13, 5.13)	4.87 (4.87, 4.87)	0.27	5.12 (5.12, 5.12)	4.90 (4.90, 4.90)	0.23	5.05 (5.05, 5.06)	4.95 (4.95, 4.95)	0.12
**TG (mmol/L)**	1.51 (1.51, 1.51)	1.33 (1.33, 1.33)	0.11	1.51 (1.51, 1.51)	1.35 (1.35, 1.35)	0.10	1.47 (1.46, 1.48)	1.39 (1.39, 1.39)	0.03
**LDL-C (mmol/L)**	2.97 (2.97, 2.97)	2.76 (2.76, 2.76)	0.28	2.96 (2.96, 2.96)	2.78 (2.78, 2.78)	0.23	2.94 (2.93, 2.95)	2.82 (2.82, 2.82)	0.16
**HDL-C (mmol/L)**	1.35 (1.34, 1.35)	1.36 (1.36, 1.36)	-0.03	1.35 (1.35, 1.35)	1.35 (1.35, 1.36)	-0.03	1.33 (1.33, 1.33)	1.35 (1.35, 1.35)	-0.07
**Hypertension, No. (%)**	1,702,579 (50.2)	1,599,178 (21.8)	0.62	1,381,995 (51.8)	1,919,762 (23.8)	0.60	36,410 (59.4)	3,265,347 (30.6)	0.60
**Diabetes, No. (%)**	537,885 (15.9)	368,887 (5.03)	0.36	446,153 (16.7)	460, 619 (5.71)	0.35	13,250 (21.6)	893,522 (8.37)	0.38
**Dyslipidemia, No. (%)**	2,287,861 (67.4)	3,985,353 (54.3)	0.27	1,789,815 (67.1)	4,483,399 (55.6)	0.24	40,110 (65.4)	6,233,104 (58.4)	0.14

Characteristics	Increased cIMT		SMD	CP		SMD	CS		SMD
	Yes	No		Yes	No		Yes	No	
**MetS, No. (%)**	1, 991,181 (58.7)	2,574,126 (35.1)	0.49	1,592,285 (59.7)	2,973,022 (36.9)	0.47	39,265 (64.0)	4,526,042 (42.4)	0.44
**Increased WBC, No. (%)**	70,791 (2.09)	126,943 (1.73)	0.03	57,256 (2.15)	140,478 (1.74)	0.03	1,697 (2.77)	196,037 (1.84)	0.06
**Increased NLR, No. (%)**	142,268 (4.19)	231,763 (3.16)	0.05	115,778 (4.34)	258,253 (3.20)	0.06	3,576 (5.83)	370,455 (3.47)	0.11
**Increased HR, No. (%)**	13,926 (0.41)	28,489 (0.39)	0.004	11,595 (0.43)	30,820 (0.38)	0.01	297 (0.48)	42,118 (0.39)	0.01

© 2024 Fu J et al. *JAMA Network Open.*

**Increased uric acid, No. (%)**	650,294 (19.2)	1,461,227 (19.9)	-0.02	512,095 (19.2)	1,599,426 (19.8)	-0.02	11,975 (19.5)	2,099,546 (19.7)	-0.004
**Increased platelet, No. (%)**	56,536 (1.67)	168,324 (2.29)	-0.05	44,004 (1.65)	180,856 (2.24)	-0.04	1,158 (1.89)	223,702 (2.10)	-0.01

BMI body mass index, CP carotid plaque, CS carotid artery stenosis, DBP diastolic blood pressure, FBG fasting blood glucose, G group, GDP gross domestic product, HDL-C high-density lipoprotein cholesterol, HR heart rate, IMT intima-media thickness, LDL-C low-density lipoprotein cholesterol, MetS Metabolic syndrome, NLR neutrophil to lymphocyte ratio, SBP systolic blood pressure, SMD standardized mean difference, TC total cholesterol, TG triglycerides, WBC white blood cells.

[a] Geometric least square mean (95% confidence interval) (all such values).

[b] Categorical variables are expressed as number (percentage) of participants.

© 2024 Fu J et al. *JAMA Network Open.*

eTable 5. Association between the risk factors as continuous variables and CAS (adjusted ORs and 95 % confidence intervals; n=10,733,975)

Characteristic	Abnormal IMT	CP	CS
	OR (95%CI) [a]	OR (95%CI)	OR (95%CI)
**Distal Factors** [b]			
**Age group (years)**	706.6 (700.2, 713.1)	504.5 (499.8, 509.3)	150.5 (144.2, 157.1)
**Sex**			
Females	1.00 (reference)	1.00 (reference)	1.00 (reference)
Males	2.152 (2.145, 2.160)	1.989 (1.982, 1.996)	2.138 (2.099, 2.177)
**Region**			
South	1.00 (reference)	1.00 (reference)	1.00 (reference)
North	1.031 (1.022, 1.040)	1.511 (1.339, 1.707)	1.559 (1.312, 1.854)
**Intermediator Factors** [c]			
**BMI group (kg/m^2)**	1.745 (1.721, 1.769)	1.295 (1.277, 1.314)	0.425 (0.396, 0.455)
**WBC (10^9/L)**	1.427 (1.417, 1.438)	1.482 (1.471, 1.493)	1.935 (1.866, 2.007)
**NLR**	1.052 (1.047, 1.057)	1.059 (1.054, 1.064)	1.144 (1.119, 1.169)
**HR (times/min)**	1.141 (1.124, 1.158)	1.325 (1.305, 1.345)	1.059 (0.983, 1.141)
**Uric acid (μmol/L)**	1.250 (1.241, 1.260)	1.315 (1.305, 1.325)	1.215 (1.170, 1.261)
**Platelet (10^9/L)**	1.070 (1.050, 1.083)	1.027 (1.020, 1.035)	0.978 (0.945, 1.012)
**Proximal Factors**			
**Hypertension** [d]			
No	1.00 (reference)	1.00 (reference)	1.00 (reference)
Yes	1.613 (1.607, 1.619)	1.635 (1.629, 1.642)	1.501 (1.473, 1.528)
**Characteristic**	**Abnormal IMT**	**CP**	**CS**

© 2024 Fu J et al. *JAMA Network Open.*

	OR (95%CI)	OR (95%CI)	OR (95%CI)
**Diabetes** [d]			
No	1.00 (reference)	1.00 (reference)	1.00 (reference)
Yes	1.633 (1.624, 1.641)	1.632 (1.623, 1.641)	1.522 (1.490, 1.555)
**Dyslipidemia** [d]			
No	1.00 (reference)	1.00 (reference)	1.00 (reference)
Yes	1.270 (1.265, 1.275)	1.228 (1.224, 1.233)	1.080 (1.060, 1.100)
**MetS** [e]			
No	1.00 (reference)	1.00 (reference)	1.00 (reference)
Yes	1.459 (1.454, 1.465)	1.478 (1.473, 1.484)	1.412 (1.386, 1.439)

BMI body mass index, cIMT carotid intima-media thickness, CP carotid plaque, CS carotid artery stenosis, HR heart rate, GDP gross domestic product, MetS metabolic syndrome, NLR neutrophil to lymphocyte ratio, WBC white blood cells.

[a] Obtained by using multivariable mixed effect logistic regression analysis.

[b] Adjusted for age, sex, GDP per capita, and region for distal factors.

[c] Adjusted for age, sex, GDP per capita, region, BMI, WBC, NLR, HR, uric acid, and platelet count for intermediator factors.

[d] Additionally adjusted for hypertension, diabetes, and dyslipidemia for proximal factors.

[e] Adjusted for age, sex, GDP per capita, region, BMI, WBC, NLR, HR, uric acid, and platelet count for MetS.

© 2024 Fu J et al. *JAMA Network Open.*

eTable 6. Association between the risk factors and CAS using multivariable logistic regression model (adjusted ORs and 95 % confidence intervals; n=10,733,975)

Characteristic	Increased cIMT OR (95%CI) [a]	CP OR (95%CI)	CS OR (95%CI)
**Distal Factors** [b]			
**Age group (years)** [f]			
20-29	0.044 (0.043, 0.044)	0.063 (0.063, 0.064)	0.155 (0.144, 0.167)
30-39	0.090 (0.090, 0.091)	0.114 (0.113, 0.114)	0.171 (0.163, 0.179)
40-49	0.305 (0.304, 0.306)	0.331 (0.329, 0.332)	0.378 (0.366, 0.390)
50-59	1.00 (reference)	1.00 (reference)	1.00 (reference)
60-~	3.929 (3.914, 3.945)	3.680 (3.666, 3.694)	3.572 (3.505, 3.640)
***P* for trend**	< 0.0001	< 0.0001	< 0.0001
**Sex**			
Females	1.00 (reference)	1.00 (reference)	1.00 (reference)
Males	2.027 (2.021, 2.034)	1.883 (1.877, 1.889)	2.176 (2.138, 2.214)
**Region**			
South	1.00 (reference)	1.00 (reference)	1.00 (reference)
North	1.718 (1.713, 1.724)	1.672 (1.666, 1.677)	1.272 (1.249, 1.294)
**Intermediator Factors** [c]			
**BMI group (kg/m^2)**			
< 18.5	0.994 (0.980, 1.007)	1.047 (1.033, 1.062)	1.311 (1.233, 1.393)
18.5-23.9	1.00 (reference)	1.00 (reference)	1.00 (reference)
24.0-27.9	1.140 (1.136, 1.144)	1.088 (1.084, 1.092)	0.933 (0.916, 0.950)
≥ 28.0	1.258 (1.252, 1.264)	1.145 (1.139, 1.150)	0.861 (0.841, 0.882)
***P* for trend**	< 0.0001	< 0.0001	< 0.0001

© 2024 Fu J et al. *JAMA Network Open.*

Characteristic	Increased cIMT<br>OR (95%CI)	CP<br>OR (95%CI)	CS<br>OR (95%CI)
**Increased WBC**	1.351 (1.336, 1.367)	1.360 (1.344, 1.376)	1.407 (1.338, 1.479)
**Increased NLR**	1.039 (1.030, 1.048)	1.065 (1.056 ,1.074)	1.200 (1.159, 1.243)
**Increased HR**	1.064 (1.037, 1.092)	1.136 (1.108, 1.166)	1.042 (0.926, 1.167)
**Increased uric acid**	1.099 (1.094, 1.103)	1.121 (1.116, 1.125)	1.146 (1.122, 1.170)
**Increased platelet**	1.121 (1.108, 1.134)	1.110 (1.097, 1.124)	1.341 (1.264, 1.423)
**Proximal Factors**			
**Hypertension** [d]			
No	1.00 (reference)	1.00 (reference)	1.00 (reference)
Yes	1.497 (1.492, 1.502)	1.503 (1.498, 1.508)	1.499 (1.473, 1.525)
**Diabetes** [d]			
No	1.00 (reference)	1.00 (reference)	1.00 (reference)
Yes	1.563 (1.555, 1.571)	1.554 (1.546, 1.562)	1.499 (1.469, 1.529)
**Dyslipidemia** [d]			
No	1.00 (reference)	1.00 (reference)	1.00 (reference)
Yes	1.241 (1.237, 1.245)	1.193 (1.189, 1.197)	1.091 (1.072, 1.110)
**MetS** [e]			
No	1.00 (reference)	1.00 (reference)	1.00 (reference)
Yes	1.422 (1.417, 1.426)	1.428 (1.403, 1.454)	1.428 (1.403, 1.454)

BMI body mass index, cIMT carotid intima-media thickness, CP carotid plaque, CS carotid artery stenosis, HR heart rate, GDP gross domestic product, MetS metabolic syndrome, NLR neutrophil to lymphocyte ratio, WBC white blood cells.

[a] Obtained by using multivariable logistic regression analysis.

[b] Adjusted for age, sex, GDP per capita, and region for distal factors.

[c] Adjusted for age, sex, GDP per capita, region, BMI, WBC, NLR, HR, uric acid, and platelet count for intermediator factors.

[d] Additionally adjusted for hypertension, diabetes, and dyslipidemia for proximal factors.

[e] Adjusted for age, sex, GDP per capita, region, BMI, WBC, NLR, HR, uric acid, and platelet count for MetS.

[f] *P* for trend < .001.

© 2024 Fu J et al. *JAMA Network Open.*

[g] P for trend > .001 and < .05.
[h] P for trend > .05.

© 2024 Fu J et al. *JAMA Network Open.*

eTable 7. Prevalence of CAS by the characteristics of study population using information from the first check-ups, 2017-2022 (n=9,847,132)

Group	All participants, No. (%)	Weighted prevalence, % (95%CI)			
		**Increased cIMT**	**CP**	**CS**	**Moderate to severe CS**
Participants	9,847,132	24.5 (23.3, 25.6)	19.5 (18.4, 20.6)	0.52 (0.35, 0.68)	0.14 (0.12, 0.16)
**Year**					
2017	664,927 (6.75)	22.8 (21.1, 24.5) [a]	17.8 (16.4, 19.3) [a]	0.44 (0.30, 0.57) [c]	0.14 (0.11, 0.17) [a]
2018	1,765,752 (17.9)	22.4 (21.2, 23.6)	16.9 (16.0, 17.9)	0.52 (0.31, 0.72)	0.13 (0.11, 0.16)
2019	2,000,426 (20.3)	24.1 (22.7, 25.4)	19.1 (17.9, 20.4)	0.63 (0.39, 0.87)	0.17 (0.15, 0.20)
2020	2,153,081 (21.9)	24.2 (22.7, 25.6)	19.5 (18.1, 21.0)	0.51 (0.24, 0.79)	0.13 (0.11, 0.15)
2021	2,384,498 (24.2)	25.8 (24.2, 27.5)	21.0 (19.3, 22.7)	0.44 (0.27, 0.60)	0.14 (0.12, 0.15)
2022	878,448 (8.92)	27.6 (25.8, 29.4)	22.1 (20.3, 24.0)	0.54 (0.29, 0.79)	0.11 (0.092, 0.12)
**Sex**					
Males	4,850,212 (54.1)	27.5 (26.4, 28.6) [a]	21.9 (20.8, 23.0) [a]	0.61 (0.48, 0.75) [b]	0.20 (0.18, 0.23) [a]
Females	4,118,472 (45.9)	21.3 (20.0, 22.5)	16.9 (15.7, 18.1)	0.42 (0.20, 0.63)	0.08 (0.07, 0.09)
**Age (years)**					
20-29	896,706 (10.0)	3.03 (2.06, 3.99) [a]	2.55 (1.61, 3.50) [a]	0.17 (0.01, 0.38) [a]	0.0009 (0.0003, 0.002) [a]
30-39	1,989,682 (22.2)	6.08 (5.00, 7.17)	4.65 (3.57, 5.73)	0.16 (0.006, 0.31)	0.016 (0.013, 0.020)
40-49	2,043,343 (22.8)	17.8 (16.7, 19.0)	12.6 (11.5, 13.7)	0.26 (0.094, 0.43)	0.002 (0.013, 0.020)
50-59	2,353,278 (26.2)	39.7 (38.4, 40.9)	29.2 (28.0, 30.5)	0.50 (0.33, 0.68)	0.095 (0.082, 0.11)
60-69	1,227,786 (13.7)	65.7 (64.6, 66.8)	53.3 (52.2, 54.4)	1.20 (0.97, 1.43)	0.42 (0.37, 0.47)
70-79	371,777 (4.15)	83.4 (82.6, 84.2)	74.0 (73.1, 75.0)	2.32 (2.03, 2.61)	0.99 (0.88, 1.09)
80-~	86,112 (0.96)	92.0 (91.5, 92.6)	86.2 (85.6, 86.9)	3.82 (3.38, 4.26)	1.68 (1.49, 1.88)

Group	All participants, No. (%)	Weighted prevalence, % (95%CI)			
		**Increased cIMT**	**CP**	**CS**	**Moderate to severe CS**
**Region (type I)**					
East	2,997,767 (27.9)	23.7 (21.2, 26.1) [a]	18.4 (15.9, 20.9) [a]	0.33 (0.22, 0.44) [b]	0.13 (0.094, 0.16) [a]
South	1,115,930 (10.4)	14.7 (13.1, 16.3)	10.8 (9.56, 12.0)	0.44 (0.08, 0.79)	0.075 (0.057, 0.092)
Central	1,649,121 (15.4)	26.3 (24.1, 28.5)	20.9 (18.9, 22.9)	0.50 (0.20, 0.79)	0.16 (0.10, 0.21)

© 2024 Fu J et al. *JAMA Network Open.*

North	1,672,581 (15.6)	29.1 (25.8, 32.4)	23.3 (20.0, 26.6)	0.66 (0.26, 1.06)	0.23 (0.18, 0.28)
Northwest	625,196 (5.82)	28.1 (25.4, 30.8)	23.9 (21.5, 26.3)	0.29 (0.17, 0.41)	0.083 (0.056, 0.11)
Southwest	955,946 (8.91)	21.5 (18.4, 24.5)	17.8 (14.8, 20.8)	0.27 (0.21, 0.34)	0.094 (0.069, 0.12)
Northeast	1,717,434 (16.0)	27.2 (24.1, 30.2)	21.7 (18.9, 24.5)	0.85 (0.54, 1.17)	0.32 (0.22, 0.42)
**Region (type Π)**					
South	3,824,949 (42.7)	19.3 (18.1, 20.6) [a]	15.0 (13.8, 16.2) [a]	0.37 (0.23, 0.51) [b]	0.080 (0.071, 0.090) [a]
North	5,143,735 (57.4)	28.7 (26.9, 30.5)	23.1 (21.3, 24.8)	0.60 (0.45, 0.74)	0.24 (0.20, 0.28)
**BMI (kg/m^2)**					
< 18.5	933,488 (10.4)	27.9 (26.2, 29.7) [a]	23.6 (21.9, 25.2) [a]	0.75 (0.55, 0.95) [a]	0.25 (0.22, 0.29) [a]
18.5-23.9	3,297,153 (36.8)	18.7 (17.5, 19.8)	15.0 (13.9, 16.1)	0.46 (0.27, 0.64)	0.11 (0.10, 0.12)
24.0-27.9	3,262,318 (36.4)	28.7 (27.6, 29.8)	22.5 (21.4, 23.7)	0.54 (0.39, 0.68)	0.15 (0.13, 0.17)
≥ 28.0	1,475,725 (16.5)	28.4 (27.2, 29.6)	21.9 (20.7, 23.1)	0.46 (0.31, 0.60)	0.12 (0.10, 0.13)
**Diabetes**					
Yes	712,980 (7.95)	56.8 (55.6, 58.0) [a]	47.5 (46.3, 48.8) [a]	1.40 (1.16, 1.64) [a]	0.52 (0.46, 0.57) [a]
No	8,255,704 (92.1)	22.3 (21.2, 23.4)	17.6 (16.5, 18.7)	0.46 (0.29, 0.62)	0.11 (0.10, 0.13)
**Hypertension**					
Yes	2,605,511 (29.1)	47.5 (46.4, 48.6) [a]	38.9 (37.8, 40.0) [a]	1.03 (0.84, 1.22) [a]	0.35 (0.32, 0.39) [a]
No	6,363,173 (71.0)	17.3 (16.1, 18.4)	13.4 (12.3, 14.6)	0.36 (0.19, 0.52)	0.07 (0.06, 0.08)
**Group**	**All participants, No. (%)**	**Weighted prevalence, % (95%CI)**			
		**Increased cIMT**	**CP**	**CS**	**Moderate to severe CS**
**Dyslipidemia**					
Yes	5,005,199 (55.8)	30.0 (28.8, 31.2) [a]	23.7 (22.5, 24.9) [a]	0.58 (0.41, 0.75) [a]	0.16 (0.14, 0.18) [a]
No	3,963,485 (44.2)	18.6 (17.5, 19.7)	15.1 (14.0, 16.1)	0.45 (0.28, 0.62)	0.12 (0.10, 0.13)
**MetS**					
Yes	1,548,988 (17.3)	36.7 (35.5, 37.9) [a]	29.4 (28.3, 30.6) [a]	0.74 (0.57, 0.91) [a]	0.24 (0.21, 0.26) [a]
No	7,419,696 (82.7)	17.3 (16.2, 18.4)	13.6 (12.5, 14.7)	0.39 (0.21, 0.56)	0.08 (0.07, 0.10)

BMI body mass index, CAS carotid atherosclerosis, cIMT carotid intima-media thickness, CP carotid plaque, CS carotid artery stenosis, MetS Metabolic syndrome.

[a] *P* for difference < .001, *P* for difference can be expressed as whether there is a statistically significant difference in prevalence between subgroups.

[b] *P* for difference > .001 and < .05.

[c] *P* for difference > .05.

© 2024 Fu J et al. *JAMA Network Open.*

eTable 8. Association between the risk factors and CAS using complete data (adjusted ORs and 95 % confidence intervals; n=7,292,261)

Characteristic	Increased cIMT OR (95%CI) [a]	CP OR (95%CI)	CS OR (95%CI)
**Distal Factors** [b]			
**Age group (years)**			
20-29	0.029 (0.029, 0.030) [f]	0.043 (0.042, 0.044) [f]	0.113 (0.101, 0.127) [f]
30-39	0.066 (0.066, 0.067)	0.082 (0.081, 0.082)	0.144 (0.135, 0.153)
40-49	0.268 (0.266, 0.269)	0.285 (0.283, 0.286)	0.323 (0.309, 0.338)
50-59	1.00 (reference)	1.00 (reference)	1.00 (reference)
60-~	3.996 (3.976, 4.016)	3.822 (3.803, 3.841)	3.643 (3.553, 3.735)
**Sex**			
Females	1.00 (reference)	1.00 (reference)	1.00 (reference)
Males	2.194 (2.185, 2.203)	2.033 (2.025, 2.042)	2.472 (2.413, 2.533)
**Region**			
South	1.00 (reference)	1.00 (reference)	1.00 (reference)
North	1.391 (1.220, 1.586)	1.545 (1.363, 1.752)	1.598 (1.335, 1.914)
**Intermediator Factors** [c]			
**BMI group (kg/m^2)**			
< 18.5	0.992 (0.975, 1.009) [f]	1.058 (1.040, 1.077) [f]	1.324 (1.225, 1.429) [f]
18.5-23.9	1.00 (reference)	1.00 (reference)	1.00 (reference)
24.0-27.9	1.129 (1.124, 1.134)	1.071 (1.066, 1.077)	0.877 (0.856, 0.898)
≥ 28.0	1.267 (1.259, 1.274)	1.145 (1.139, 1.152)	0.796 (0.771, 0.821)
**Increased WBC**	1.343 (1.323, 1.363)	1.348 (1.328, 1.369)	1.385 (1.296, 1.477)
**Increased NLR**	1.069 (1.058, 1.080)	1.092 (1.081 ,1.104)	1.251 (1.197, 1.307)
**Increased HR**	1.200 (1.166, 1.234)	1.283 (1.247, 1.320)	1.171 (1.033, 1.322)

© 2024 Fu J et al. *JAMA Network Open.*

Characteristic	Increased cIMT OR (95%CI) [a]	CP OR (95%CI)	CS OR (95%CI)
**Increased uric acid**	1.131 (1.125, 1.137)	1.162 (1.156, 1.168)	1.213 (1.180, 1.245)
**Increased platelet**	1.062 (1.051, 1.073)	1.109 (1.092, 1.127)	1.322 (1.223, 1.427)
**Proximal Factors**			
**Hypertension** [d]			
No	1.00 (reference)	1.00 (reference)	1.00 (reference)
Yes	1.677 (1.670, 1.685)	1.716 (1.709, 1.724)	1.638 (1.599, 1.678)
**Diabetes** [d]			
No	1.00 (reference)	1.00 (reference)	1.00 (reference)
Yes	1.639 (1.628, 1.649)	1.643 (1.633, 1.654)	1.565 (1.524, 1.606)
**Dyslipidemia** [d]			
No	1.00 (reference)	1.00 (reference)	1.00 (reference)
Yes	1.272 (1.266, 1.277)	1.224 (1.218, 1.230)	1.066 (1.041, 1.091)
**MetS** [e]			
No	1.00 (reference)	1.00 (reference)	1.00 (reference)
Yes	1.474 (1.468, 1.481)	1.487 (1.480, 1.494)	1.401 (1.368, 1.435)

BMI body mass index, cIMT carotid intima-media thickness, CP carotid plaque, CS carotid artery stenosis, GDP gross domestic product, HR heart rate, MetS metabolic syndrome, NLR neutrophil to lymphocyte ratio, WBC white blood cells.

[a] Obtained by using multivariable mixed effect logistic regression analysis.

[b] Adjusted for age, sex, GDP per capita, and region for distal factors.

[c] Adjusted for age, sex, GDP per capita, region, BMI, WBC, NLR, HR, uric acid, and platelet count for intermediator factors.

[d] Additionally adjusted for hypertension, diabetes, and dyslipidemia for proximal factors.

[e] Adjusted for age, sex, GDP per capita, region, BMI, WBC, NLR, HR, uric acid, and platelet count for MetS.

[f] *P* for trend < .001.

[g] *P* for trend > .001 and < .05.

[h] *P* for trend > .05.

© 2024 Fu J et al. *JAMA Network Open.*

eTable 9. Association between the risk factors and CAS using full model (adjusted ORs and 95 % confidence intervals; n=10,733,975)

Characteristic	Increased cIMT OR (95%CI) [a]	CP OR (95%CI)	CS OR (95%CI)
**Distal Factors** [b]			
**Age group (years)**			
20-29	0.036 (0.035, 0.036)	0.051 (0.051, 0.052)	0.183 (0.170, 0.197)
30-39	0.079 (0.078, 0.079)	0.096 (0.095, 0.097)	0.200 (0.191, 0.210)
40-49	0.292 (0.291, 0.294)	0.312 (0.311, 0.314)	0.413 (0.400, 0.427)
50-59	1.00 (reference)	1.00 (reference)	1.00 (reference)
60-~	3.924 (3.908, 3.941)	3.738 (3.723, 3.754)	3.253 (3.191, 3.316)
***P* for trend**	< 0.0001	< 0.0001	< 0.0001
**Sex**			
Females	1.00 (reference)	1.00 (reference)	1.00 (reference)
Males	1.961 (1.954, 1.968)	1.851 (1.844, 1.857)	2.114 (2.077, 2.152)
**Region**			
South	1.00 (reference)	1.00 (reference)	1.00 (reference)
North	1.334 (1.178, 1.512)	1.512 (1.344, 1.701)	1.284 (1.261, 1.308)
**Intermediator Factors** [c]			
**BMI group (kg/m^2)**			
< 18.5	1.070 (1.054, 1.085)	1.139 (1.122, 1.156)	1.431 (1.345, 1.520)
18.5-23.9	1.00 (reference)	1.00 (reference)	1.00 (reference)
24.0-27.9	1.024 (1.020, 1.028)	0.975 (0.972, 0.979)	0.843 (0.827, 0.859)
≥ 28.0	1.004 (0.999, 1.009)	0.913 (0.908, 0.918)	0.708 (0.691, 0.726)
***P* for trend**	< 0.01	< 0.0001	< 0.0001

© 2024 Fu J et al. *JAMA Network Open.*

Characteristic	Increased cIMT<br>OR (95%CI) [a]	CP<br>OR (95%CI)	CS<br>OR (95%CI)
**Increased WBC**	1.239 (1.224, 1.255)	1.255 (1.239, 1.271)	1.330 (1.265, 1.398)
**Increased NLR**	1.047 (1.038, 1.056)	1.062 (1.053 ,1.072)	1.159 (1.119, 1.201)
**Increased HR**	0.953 (0.928, 0.979)	1.023 (0.995, 1.051)	0.898 (0.799, 1.006)
**Increased uric acid**	1.052 (1.048, 1.057)	1.082 (1.077, 1.087)	1.107 (1.084, 1.130)
**Increased platelet**	1.064 (1.051, 1.077)	1.073 (1.059, 1.087)	1.317 (1.240, 1.396)
**Proximal Factors**			
**Hypertension** [d]			
No	1.00 (reference)	1.00 (reference)	1.00 (reference)
Yes	1.542 (1.536, 1.547)	1.557 (1.551, 1.563)	1.439 (1.413, 1.466)
**Diabetes** [d]			
No	1.00 (reference)	1.00 (reference)	1.00 (reference)
Yes	1.557 (1.549, 1.566)	1.553 (1.544, 1.561)	1.440 (1.410, 1.470)
**Dyslipidemia** [d]			
No	1.00 (reference)	1.00 (reference)	1.00 (reference)
Yes	1.221 (1.217, 1.226)	1.172 (1.167, 1.176)	1.056 (1.038, 1.075)
**MetS** [e]			
No	1.00 (reference)	1.00 (reference)	1.00 (reference)
Yes	1.139 (1.135, 1.144)	1.155 (1.150, 1.160)	1.153 (1.130, 1.176)

BMI body mass index, cIMT carotid intima-media thickness, CP carotid plaque, CS carotid artery stenosis, GDP gross domestic product, HR heart rate, MetS metabolic syndrome, NLR neutrophil to lymphocyte ratio, WBC white blood cells.

[a] Obtained by using multivariable mixed effect logistic regression analysis.

[b] Adjusted for age, sex, GDP per capita, region, BMI, WBC, NLR, HR, uric acid, platelet count, hypertension, diabetes, dyslipidemia, and MetS.

[c] Adjusted for age, sex, GDP per capita, region, BMI, WBC, NLR, HR, uric acid, and platelet count for intermediator factors.

[d] Additionally adjusted for hypertension, diabetes, and dyslipidemia for proximal factors.

[e] Adjusted for age, sex, GDP per capita, region, BMI, WBC, NLR, HR, uric acid, and platelet count for MetS.

[f] *P* for trend < .001.

© 2024 Fu J et al. *JAMA Network Open.*

[g] *P* for trend > .001 and < .05.
[h] *P* for trend > .05.

© 2024 Fu J et al. *JAMA Network Open.*

eTable 10. Association between the risk factors and CAS (adjusted ORs and 95 % confidence intervals; n=10,733,975)

Characteristic	Increased cIMT OR (95%CI) [a]	CP OR (95%CI)	CS OR (95%CI)
**Distal Factors** [b]			
**Age group (years)**			
20-29	0.028 (0.027, 0.028) [f]	0.040 (0.040, 0.041) [f]	0.161 (0.150, 0.173) [f]
30-39	0.065 (0.065, 0.066)	0.079 (0.079, 0.080)	0.175 (0.168, 0.184)
40-49	0.266 (0.265, 0.268)	0.284 (0.283, 0.285)	0.367 (0.355, 0.379)
50-59	1.00 (reference)	1.00 (reference)	1.00 (reference)
60-~	4.270 (4.252, 4.288)	4.065 (4.048, 4.081)	3.849 (3.774, 3.925)
***P* for trend**	< 0.0001	< 0.0001	< 0.0001
**Sex**			
Females	1.00 (reference)	1.00 (reference)	1.00 (reference)
Males	2.039 (2.025, 2.052)	1.894 (1.881, 1.907)	3.443 (3.339, 3.549)
**Region**			
South	1.00 (reference)	1.00 (reference)	1.00 (reference)
North	1.676 (1.440, 1.951)	1.704 (1.484, 1.956)	1.540 (1.289, 1.841)
**Intermediator Factors**[c]			
**BMI group (kg/m^2)**			
< 18.5	0.982 (0.968, 0.996) [f]	1.043 (1.027, 1.058) [f]	1.335 (1.252, 1.422) [f]
18.5-23.9	1.00 (reference)	1.00 (reference)	1.00 (reference)
24.0-27.9	1.156 (1.152, 1.161)	1.103 (1.098, 1.107)	0.925 (0.908, 0.943)
≥ 28.0	1.285 (1.279, 1.292)	1.168 (1.162, 1.174)	0.833 (0.812, 0.854)
***P* for trend**	< 0.0001	< 0.0001	< 0.0001

© 2024 Fu J et al. *JAMA Network Open.*

Characteristic	Increased cIMT OR (95%CI)	CP OR (95%CI)	CS OR (95%CI)
**Increased WBC**	1.348 (1.332, 1.365)	1.359 (1.342, 1.376)	1.408 (1.336, 1.483)
**Increased NLR**	1.065 (1.056, 1.075)	1.088 (1.079 ,1.098)	1.191 (1.148, 1.235)
**Increased HR**	1.167 (1.136, 1.198)	1.246 (1.213, 1.281)	1.032 (0.914, 1.165)
**Increased uric acid**	1.126 (1.121, 1.131)	1.152 (1.147, 1.157)	1.129 (1.105, 1.153)
**Increased platelet**	1.098 (1.084, 1.111)	1.108 (1.094, 1.123)	1.271 (1.194, 1.352)
**Proximal Factors**			
**Hypertension** [d]			
No	1.00 (reference)	1.00 (reference)	1.00 (reference)
Yes	1.605 (1.599, 1.611)	1.624 (1.618, 1.630)	1.486 (1.459, 1.513)
**Diabetes** [d]			
No	1.00 (reference)	1.00 (reference)	1.00 (reference)
Yes	1.618 (1.609, 1.627)	1.618 (1.609, 1.626)	1.504 (1.473, 1.536)
**Dyslipidemia** [d]			
No	1.00 (reference)	1.00 (reference)	1.00 (reference)
Yes	1.267 (1.262, 1.271)	1.218 (1.213, 1.222)	1.059 (1.040, 1.078)
**MetS** [e]			
No	1.00 (reference)	1.00 (reference)	1.00 (reference)
Yes	1.482 (1.477, 1.487)	1.495 (1.489, 1.500)	1.411 (1.386, 1.438)

BMI body mass index, cIMT carotid intima-media thickness, CP carotid plaque, CS carotid artery stenosis, GDP gross domestic product, HR heart rate, MetS metabolic syndrome, NLR neutrophil to lymphocyte ratio, WBC white blood cells.

[a] Obtained by using multivariable mixed effect logistic regression analysis.

[b] Adjusted for age, sex, GDP per capita, and region for distal factors.

[c] Adjusted for age, sex, GDP per capita, region, BMI, WBC, NLR, HR, uric acid, platelet count, province-level average educational years, smoking prevalence, and drinking prevalence for intermediator factors; Risk factors that were included formed a two-level hierarchical structure, with participants at level one and centers at level two. age, sex, GDP per capita, region, BMI, WBC, NLR, HR, uric acid, and platelet count were included at the participant level with centers as the random effect, while regional average educational years, smoking prevalence, and drinking prevalence were further included at the center level.

© 2024 Fu J et al. *JAMA Network Open.*

[d] Additionally adjusted for hypertension, diabetes, and dyslipidemia for proximal factors.
[e] Adjusted for age, sex, GDP per capita, region, BMI, WBC, NLR, HR, uric acid, platelet count, province-level average educational years, smoking prevalence, and drinking prevalence for MetS.
[f] *P* for trend < .001.
[g] *P* for trend > .001 and < .05.
[h] *P* for trend > .05.

© 2024 Fu J et al. *JAMA Network Open.*

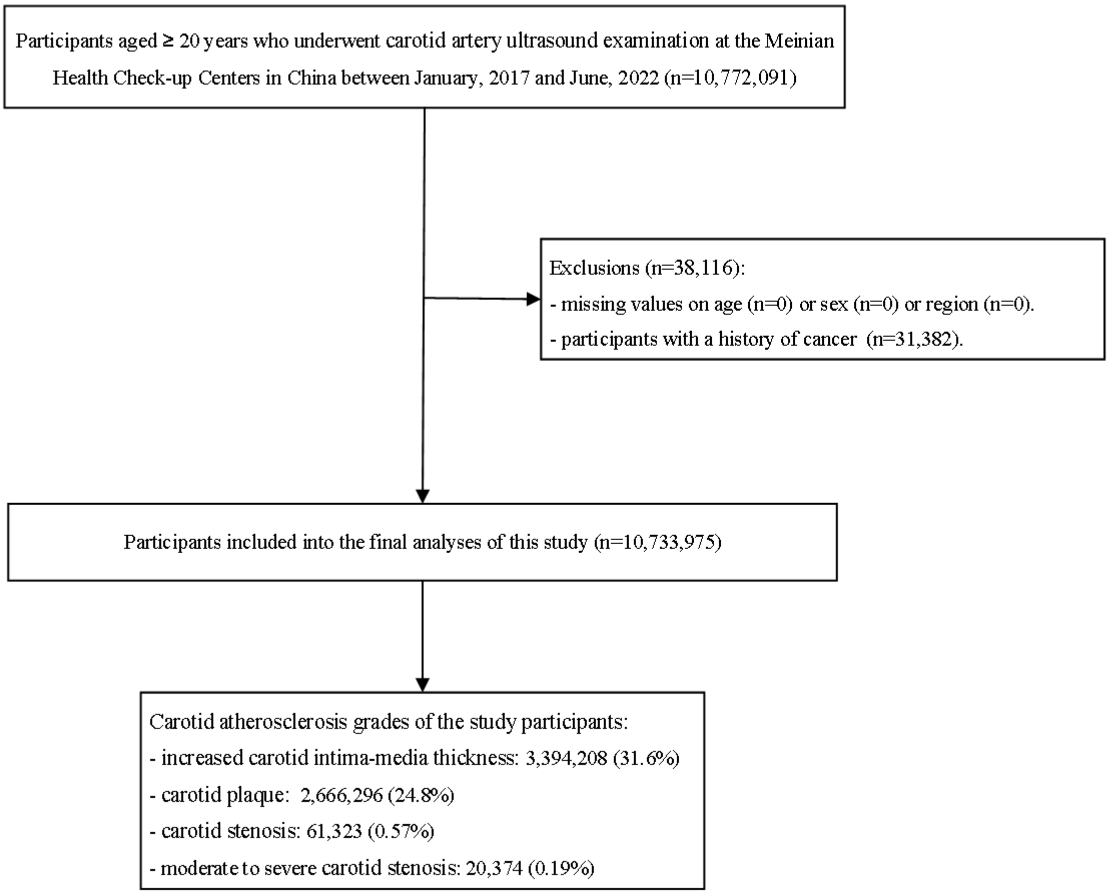

eFigure 1. Flowchart of the study participation.

© 2024 Fu J et al. *JAMA Network Open.*

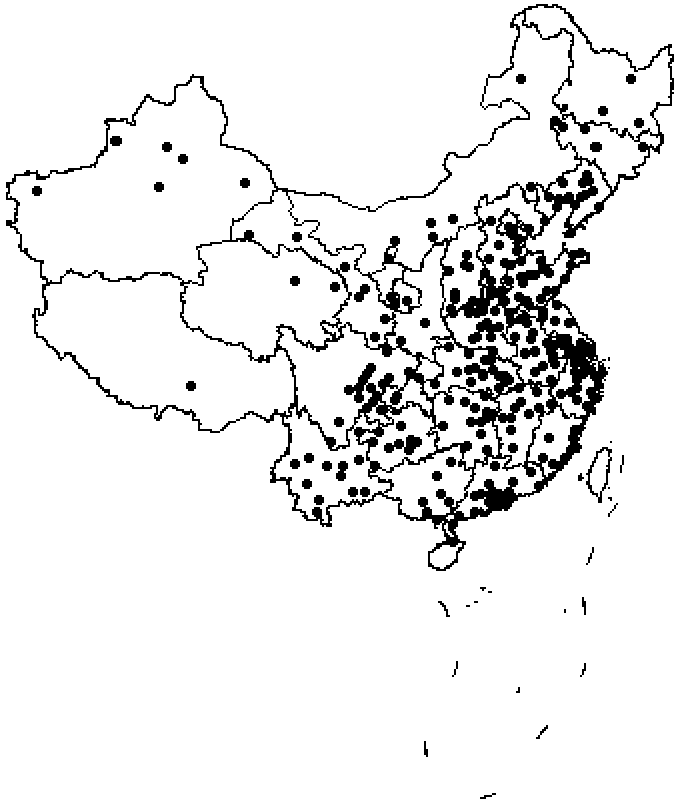

eFigure 2. Geographical distribution of the check-up centers included into the study.

© 2024 Fu J et al. *JAMA Network Open.*

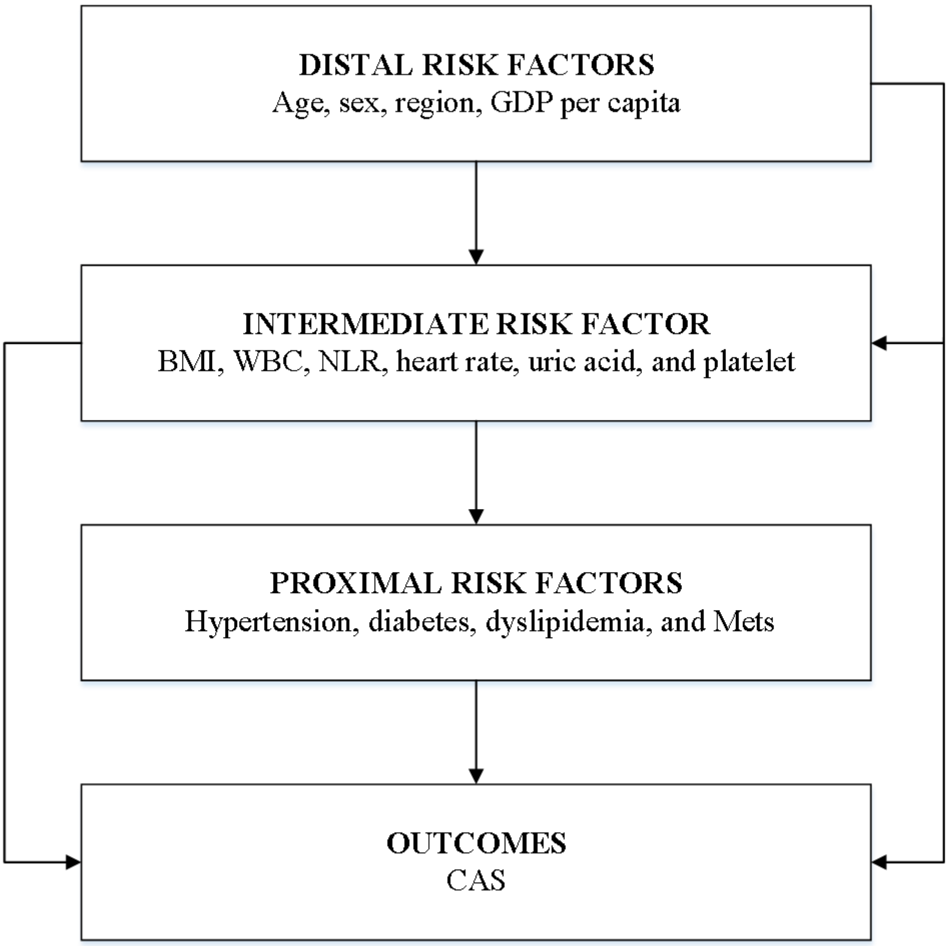

eFigure 3. Conceptual model risk factors for CAS. BMI body mass index, CAS carotid atherosclerosis, GDP gross domestic product, Mets metabolic syndrome, NLR neutrophil to lymphocyte ratio, WBC white blood cells.

© 2024 Fu J et al. *JAMA Network Open.*

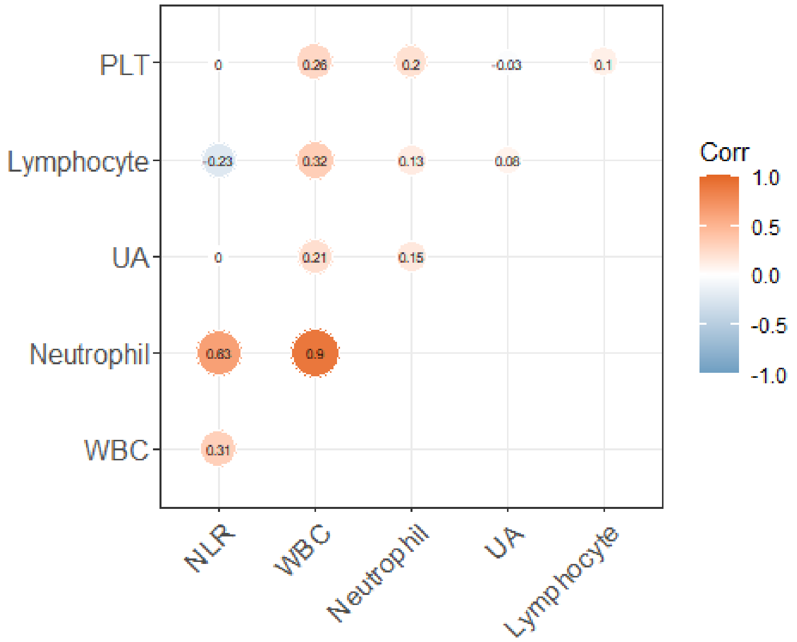

eFigure 4. Correlations between participants' blood relevant parameters. NLR neutrophil to lymphocyte ratio, PLT platelet count, UA uric acid, WBC white blood cells.

© 2024 Fu J et al. *JAMA Network Open.*

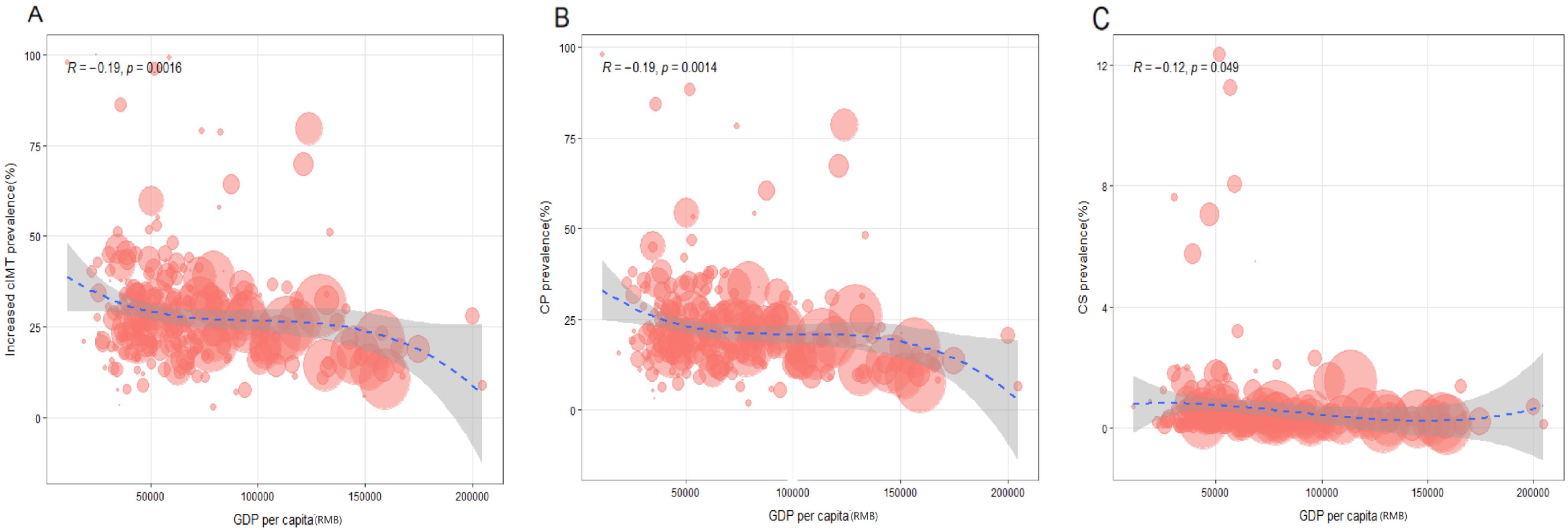

eFigure 5. Association between prevalence of different grades of CAS, and GDP per capita. Each bubble in the figure represents the prevalence of each city, and the size of the bubble reflects the sample size of the city. The dotted lines reflect the associations between the prevalence and GDP per capita, and the gray shadows represent the corresponding 95% confidence intervals.

© 2024 Fu J et al. *JAMA Network Open.*

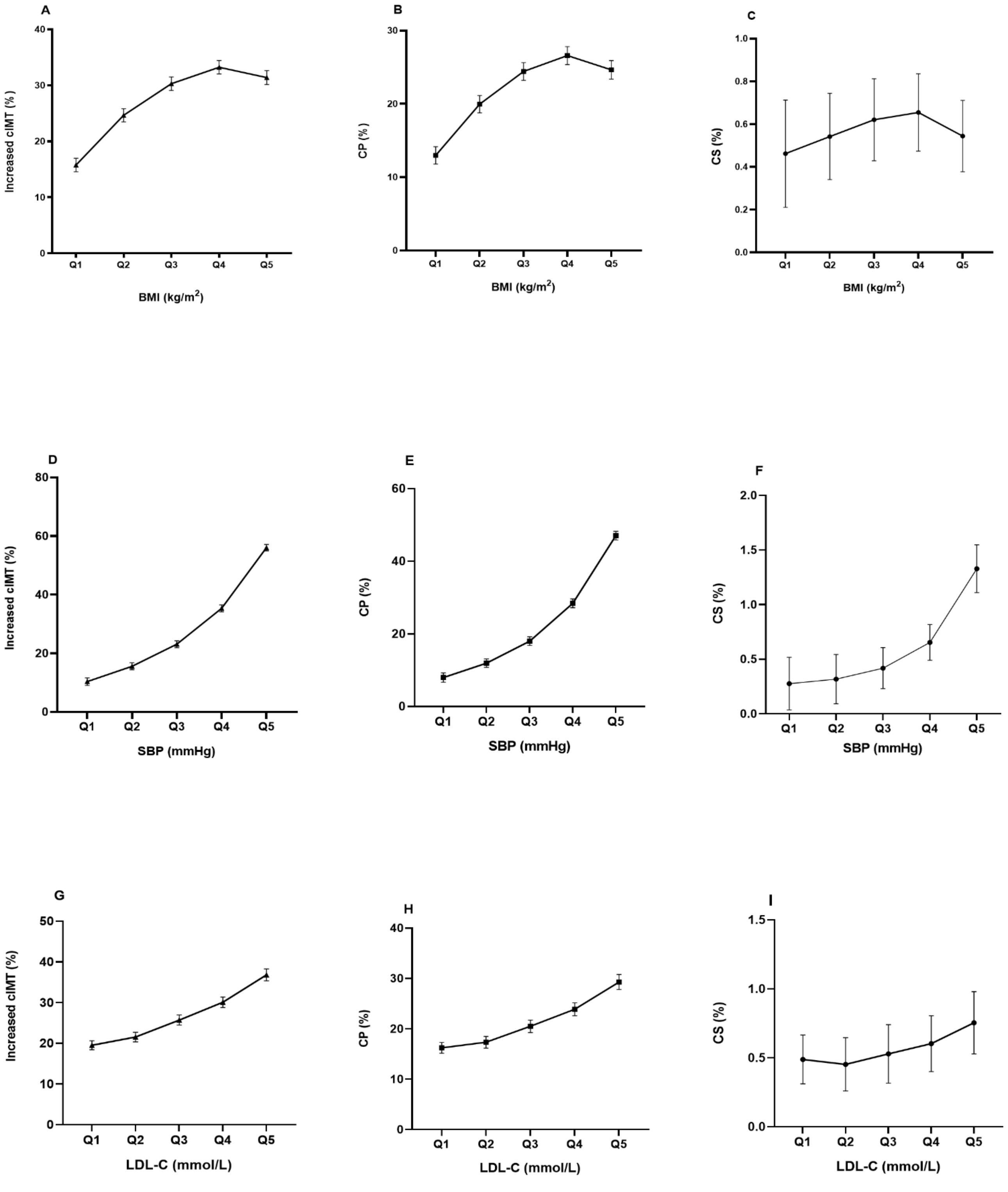

eFigure 6. Prevalence of increased cIMT, CP and CS by BMI, SBP, and LDL-C quintiles after age, gender, and regional standardization. BMI body mass index, CAS carotid atherosclerosis, cIMT carotid intima-media thickness, CP carotid plaque, LDL-C low-density lipoprotein-cholesterol, Q quintiles, SBP systolic blood pressure.

© 2024 Fu J et al. *JAMA Network Open.*

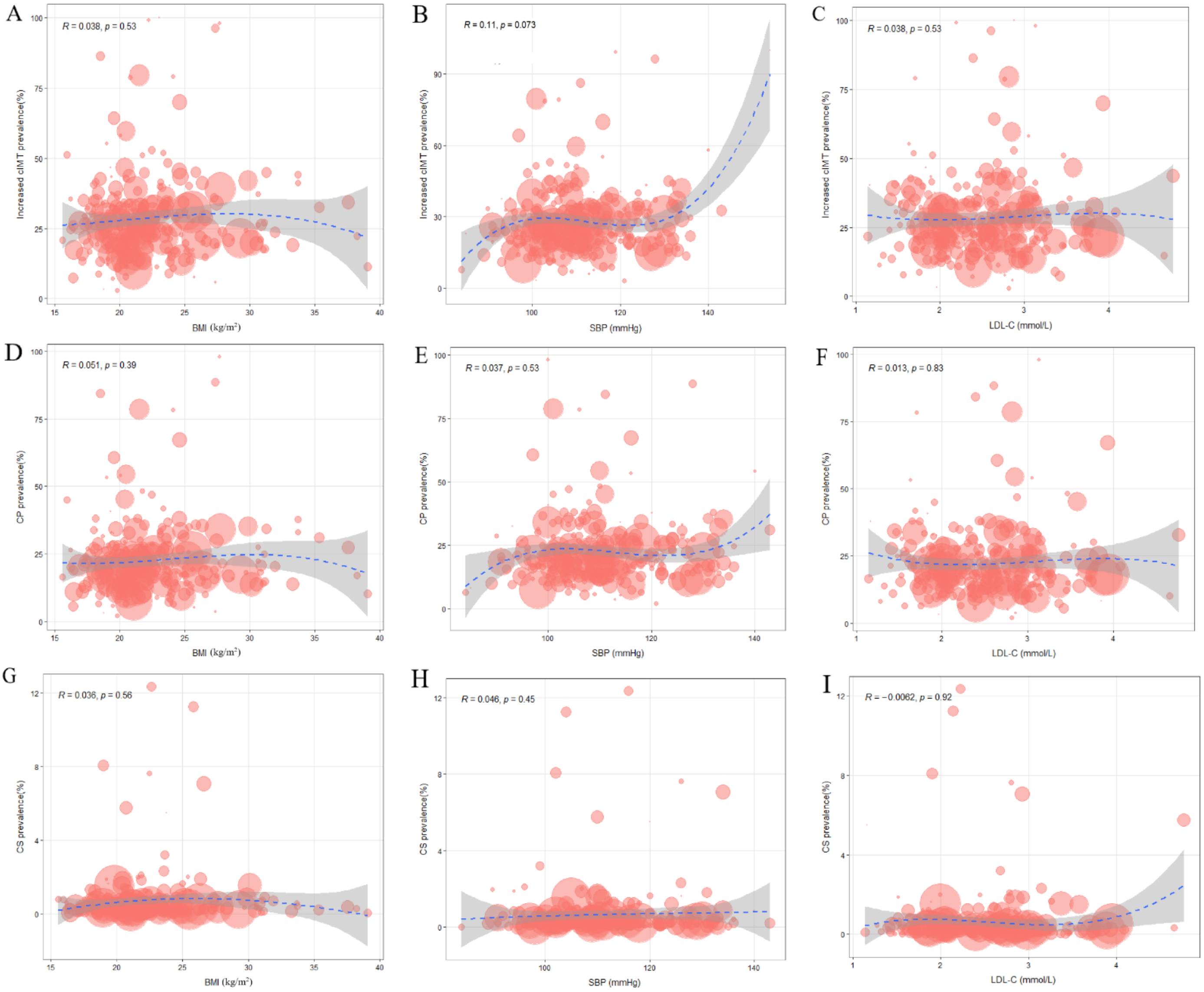

eFigure 7. Association between prevalence of different grades of CAS and BMI, SBP, and LDL-C levels. Each bubble in the figure represents the prevalence of each city, and the size of the bubble reflects the sample size of the city. The dotted lines reflect the associations between the prevalence and BMI, SBP, and LDL-C levels, and the gray shadows represent the corresponding 95% confidence intervals. BMI body mass index, CAS carotid atherosclerosis, cIMT carotid intima-media thickness, CP carotid plaque, LDL-C low-density lipoprotein-cholesterol, SBP systolic blood pressure.

© 2024 Fu J et al. *JAMA Network Open.*

A Relative contribution increased cIMT across province

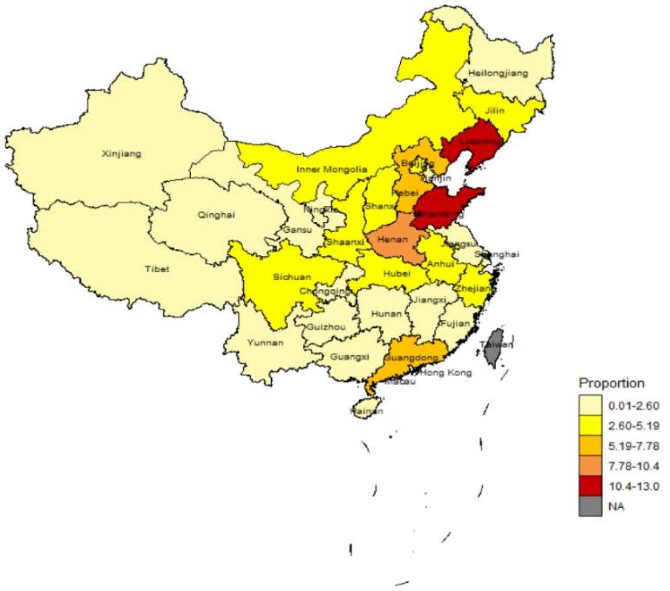

B Relative contribution CP across province

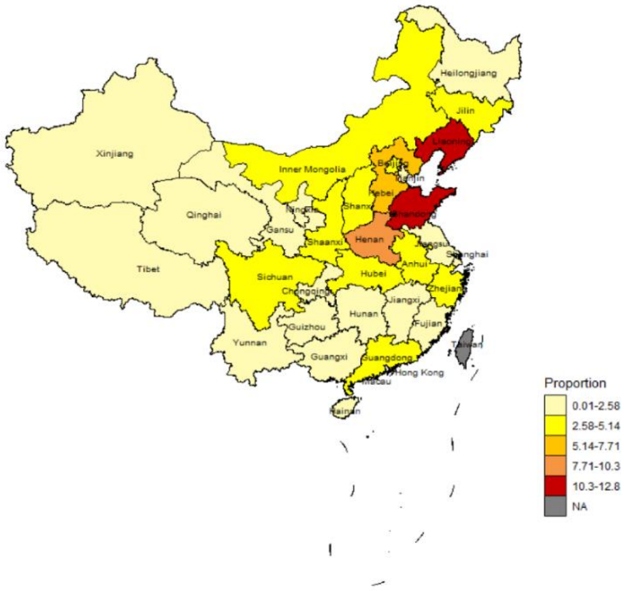

C Relative contribution CS across province

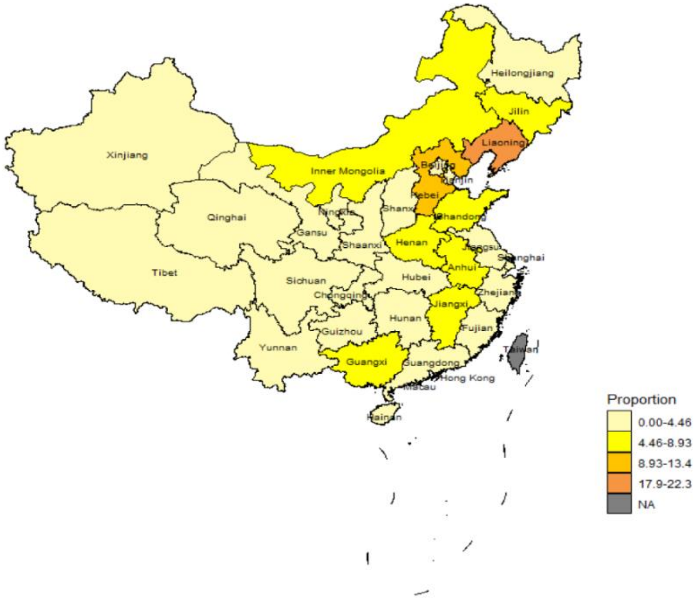

k Open.

eFigure 8. Relative contribution to the total burden of CAS by region. Age- and sex- standardized prevalence of CAS was calculated for each province according to the 2010 National Population Census. CAS carotid atherosclerosis, CP carotid plaque, cIMT carotid intima-media thickness

© 2024 Fu J et al. *JAMA Network Open.*

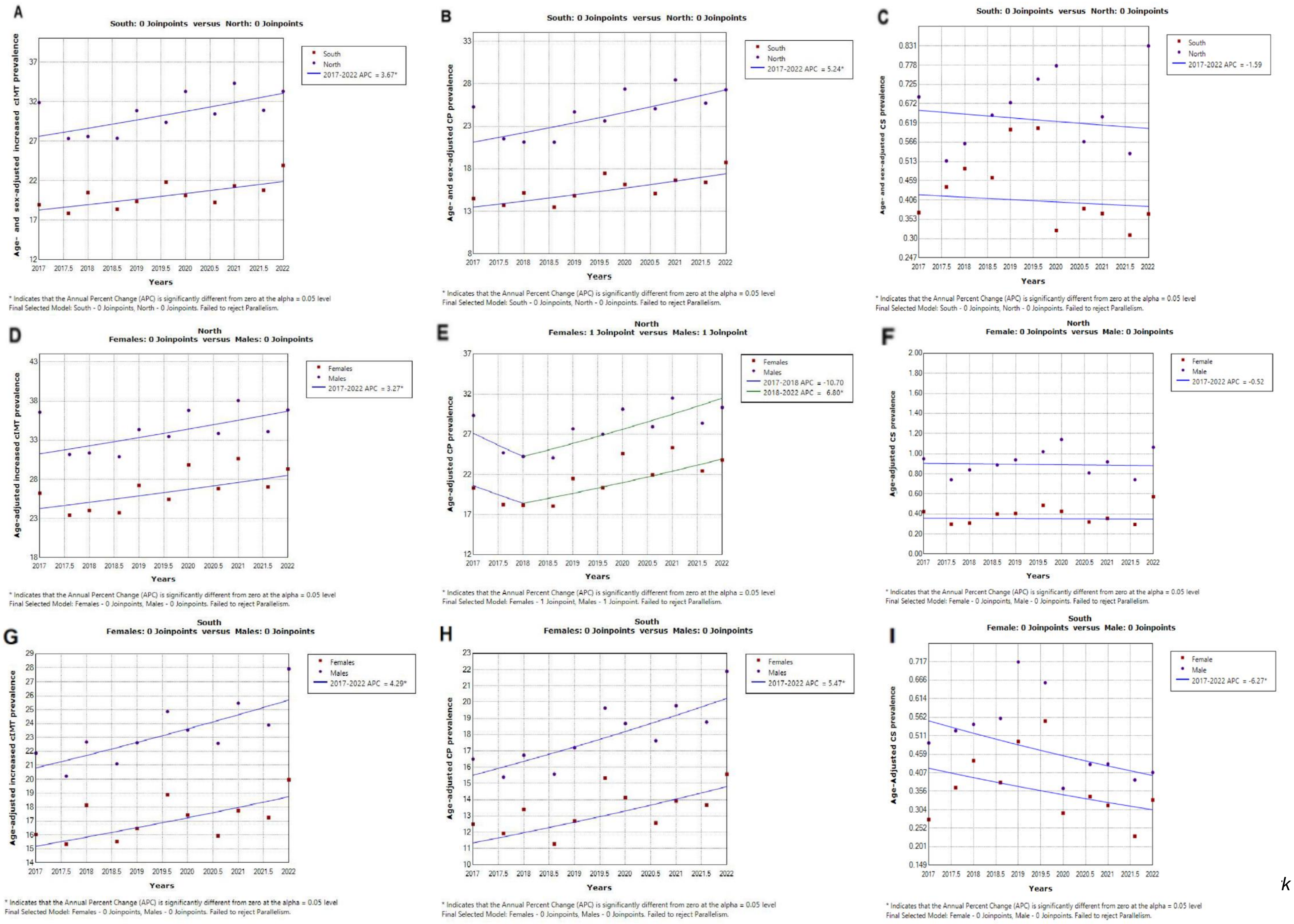
A
South: 0 Joinpoints versus North: 0 Joinpoints
South
North
2017-2022 APC = 3.67*
Age- and sex-adjusted increased cIMT prevalence
Years
* Indicates that the Annual Percent Change (APC) is significantly different from zero at the alpha = 0.05 level
Final Selected Model: South - 0 Joinpoints, North - 0 Joinpoints. Failed to reject Parallelism.
B
South: 0 Joinpoints versus North: 0 Joinpoints
South
North
2017-2022 APC = 5.24*
Age- and sex-adjusted CP prevalence
Years
* Indicates that the Annual Percent Change (APC) is significantly different from zero at the alpha = 0.05 level
Final Selected Model: South - 0 Joinpoints, North - 0 Joinpoints. Failed to reject Parallelism.
C
South: 0 Joinpoints versus North: 0 Joinpoints
South
North
2017-2022 APC = -1.59
Age- and sex-adjusted CS prevalence
Years
* Indicates that the Annual Percent Change (APC) is significantly different from zero at the alpha = 0.05 level
Final Selected Model: South - 0 Joinpoints, North - 0 Joinpoints. Failed to reject Parallelism.
D
North
Females: 0 Joinpoints versus Males: 0 Joinpoints
Females
Males
2017-2022 APC = 3.27*
Age-adjusted increased cIMT prevalence
Years
* Indicates that the Annual Percent Change (APC) is significantly different from zero at the alpha = 0.05 level
Final Selected Model: Females - 0 Joinpoints, Males - 0 Joinpoints. Failed to reject Parallelism.
E
North
Females: 1 Joinpoint versus Males: 1 Joinpoint
Females
Males
2017-2018 APC = -10.70
2018-2022 APC = 6.80*
Age-adjusted CP prevalence
Years
* Indicates that the Annual Percent Change (APC) is significantly different from zero at the alpha = 0.05 level
Final Selected Model: Females - 1 Joinpoint, Males - 1 Joinpoint. Failed to reject Parallelism.
F
North
Female: 0 Joinpoints versus Male: 0 Joinpoints
Female
Male
2017-2022 APC = -0.52
Age-adjusted CS prevalence
Years
* Indicates that the Annual Percent Change (APC) is significantly different from zero at the alpha = 0.05 level
Final Selected Model: Female - 0 Joinpoints, Male - 0 Joinpoints. Failed to reject Parallelism.
G
South
Females: 0 Joinpoints versus Males: 0 Joinpoints
Females
Males
2017-2022 APC = 4.29*
Age-adjusted increased cIMT prevalence
Years
* Indicates that the Annual Percent Change (APC) is significantly different from zero at the alpha = 0.05 level
Final Selected Model: Females - 0 Joinpoints, Males - 0 Joinpoints. Failed to reject Parallelism.
H
South
Females: 0 Joinpoints versus Males: 0 Joinpoints
Females
Males
2017-2022 APC = 5.47*
Age-adjusted CP prevalence
Years
* Indicates that the Annual Percent Change (APC) is significantly different from zero at the alpha = 0.05 level
Final Selected Model: Females - 0 Joinpoints, Males - 0 Joinpoints. Failed to reject Parallelism.
I
South
Female: 0 Joinpoints versus Male: 0 Joinpoints
Female
Male
2017-2022 APC = -6.27*
Age-Adjusted CS prevalence
Years
* Indicates that the Annual Percent Change (APC) is significantly different from zero at the alpha = 0.05 level
Final Selected Model: Female - 0 Joinpoints, Male - 0 Joinpoints. Failed to reject Parallelism.

eFigure 9. Joinpoint regression analysis of the trend for the prevalence of CAS in the South and North populations from 2017 to 2022. * Indicates that the Annual Percent Change (APC) is significantly different from zero at the alpha=0.05 level. Failed to reject Parallelism. CAS carotid atherosclerosis, CP carotid plaque, cIMT carotid intima-media thickness.

© 2024 Fu J et al. *JAMA Network Open.*

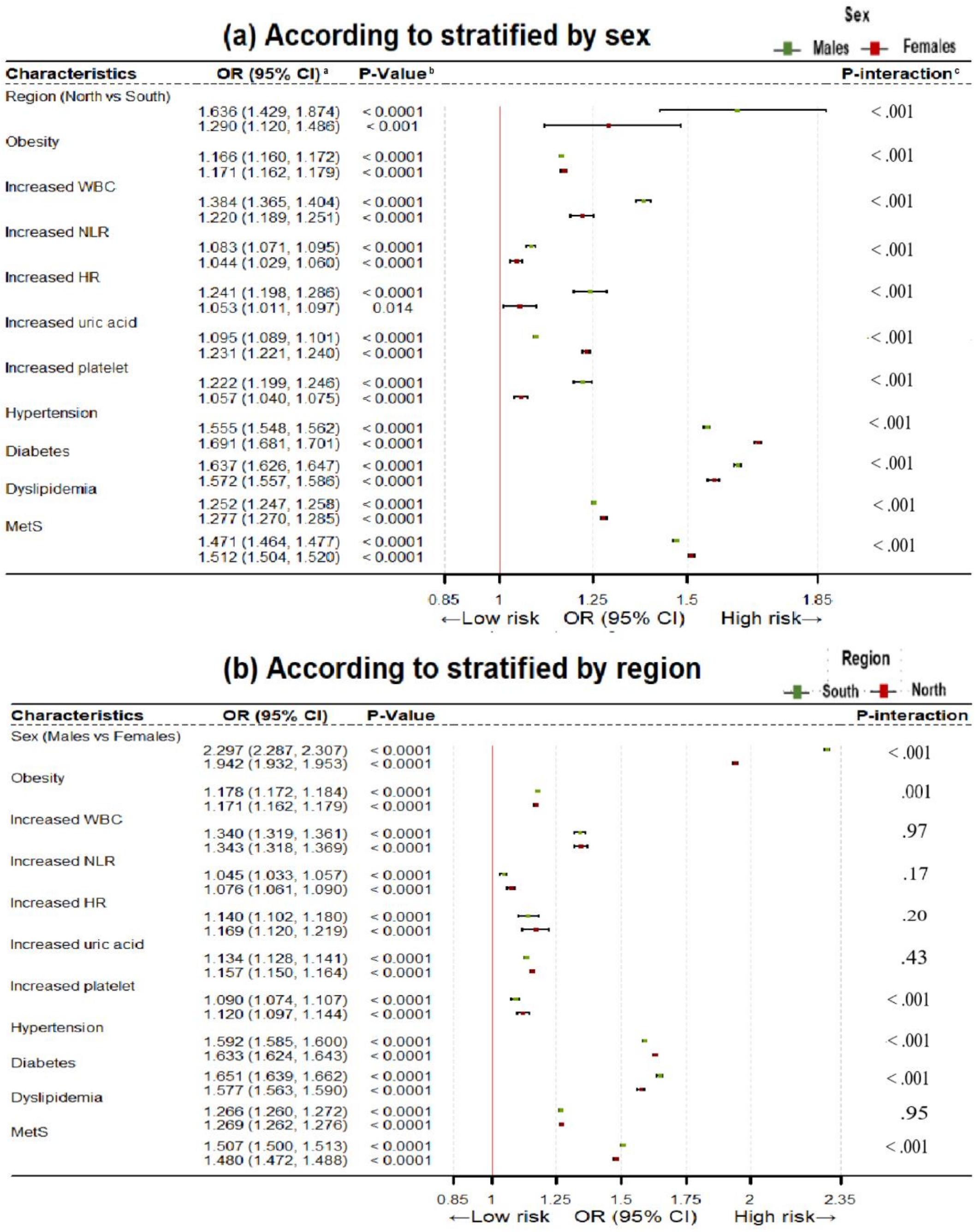

eFigure 10. Association between the risk factors and increased cIMT, stratified by sex and region. BMI body mass index, cIMT carotid intima-media thickness, HR heart rate, MetS Metabolic syndrome, NLR neutrophil to lymphocyte ratio, WBC white blood cells. [a] Adjustment factors were determined by the conceptual framework. [b] Obtained using a multivariable mixed effect logistic regression analysis. [c] Comparison between the odds ratios associated with sex (A) or region (B) using *P* for multiplicative interaction.

© 2024 Fu J et al. *JAMA Network Open.*

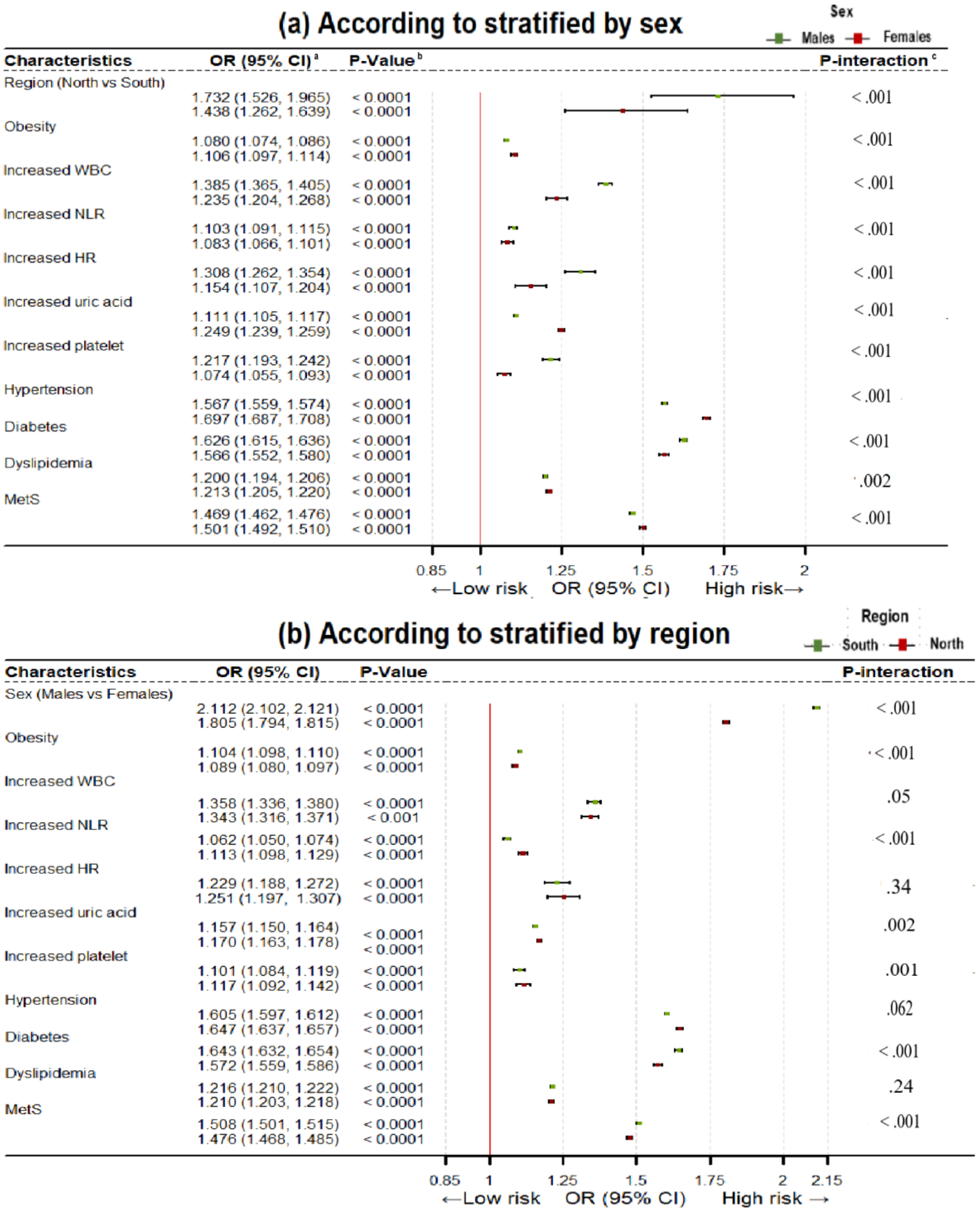

eFigure 11. Association between the risk factors and CP, stratified by sex and region. BMI body mass index, CP carotid plaque, HR heart rate, MetS Metabolic syndrome, NLR neutrophil to lymphocyte ratio, WBC white blood cells. [a] Adjustment factors were determined using a conceptual framework. [b] Obtained using multivariable mixed-effects logistic regression analysis. [c] Comparison between odds ratios associated with sex (A) or region (B) using *P* for multiplicative interactions.

© 2024 Fu J et al. *JAMA Network Open.*

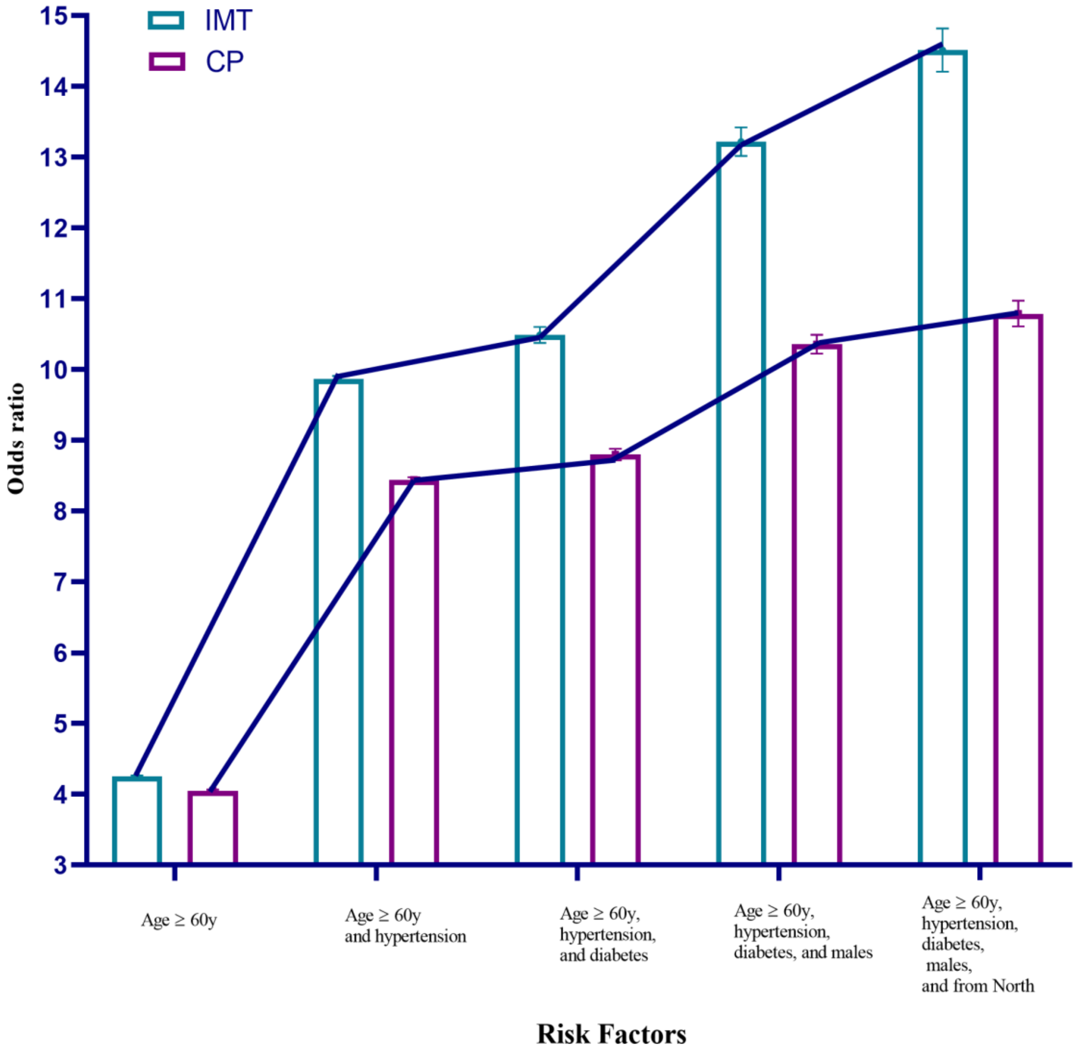

eFigure 12. Additive risk plot showing odds ratios for increased cIMT and CP when considering multiple risk factors simultaneously. CP carotid plaque, cIMT carotid intima-media thickness.

© 2024 Fu J et al. *JAMA Network Open.*

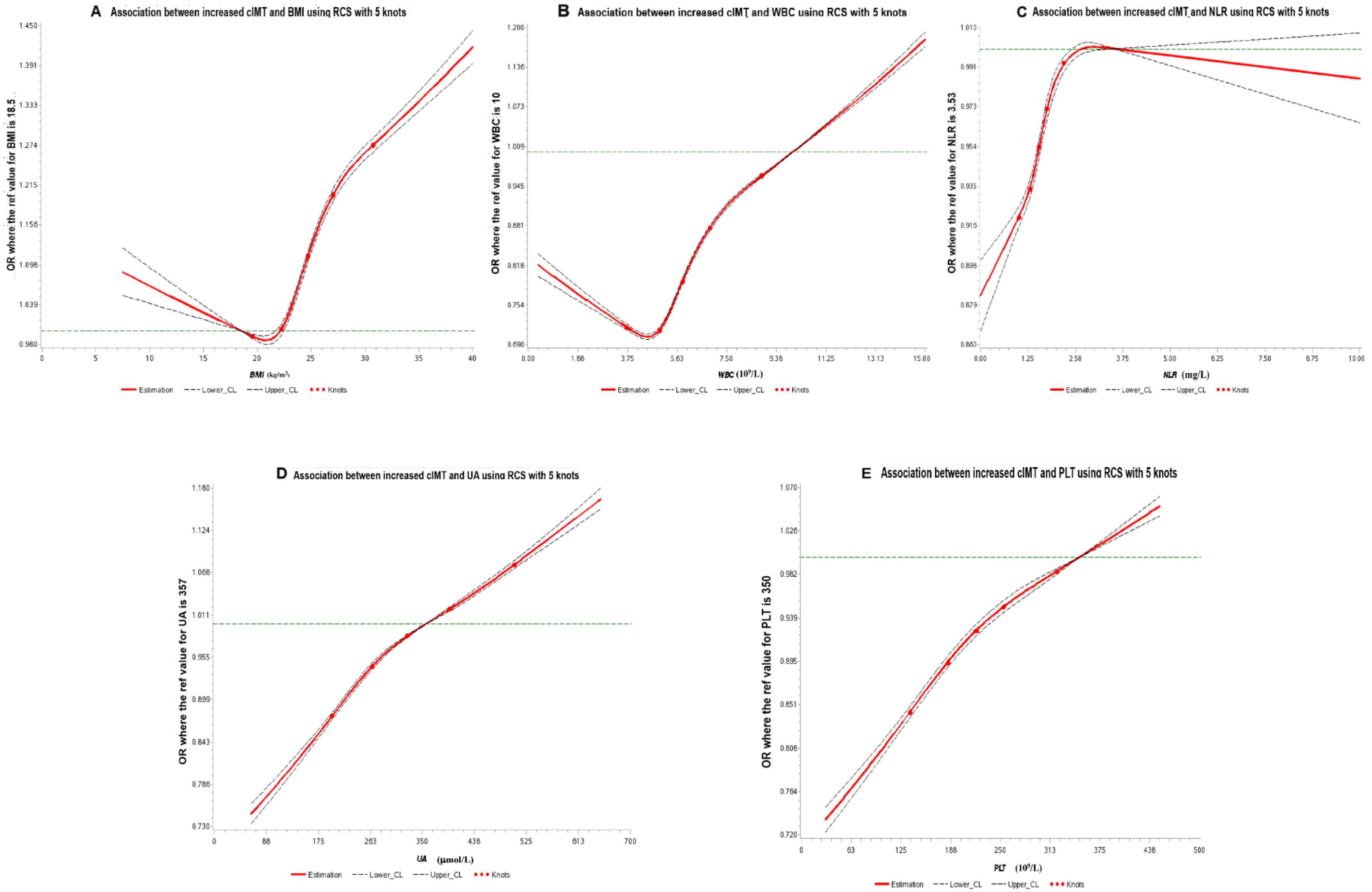

© 2024 Fu J et al. *JAMA Network Open.*

eFigure 13. The restricted cubic spline for the association between risk factors (A-E) and increased cIMT in the overall population. Solid and dashed lines represent OR and 95% CI based on restricted cubic splines in the logistic regression model. Horizontal dashed line represents the reference value. Knots were placed at the 5th, 25th, 50th, 75th and 95th percentiles of the risk factors distribution, and the reference values were listed to the left of the Y axis for all variables, e.g., the reference value of BMI as 18.5 kg/m^2. Adjustment factors were age, sex, GDP per capita, region, BMI, WBC, NLR, HR, UA, and PLT. BMI body mass index, cIMT carotid intima-media thickness, GDP gross domestic product, NLR neutrophil to lymphocyte ratio, PLT platelet count, UA uric acid, WBC white blood cells.

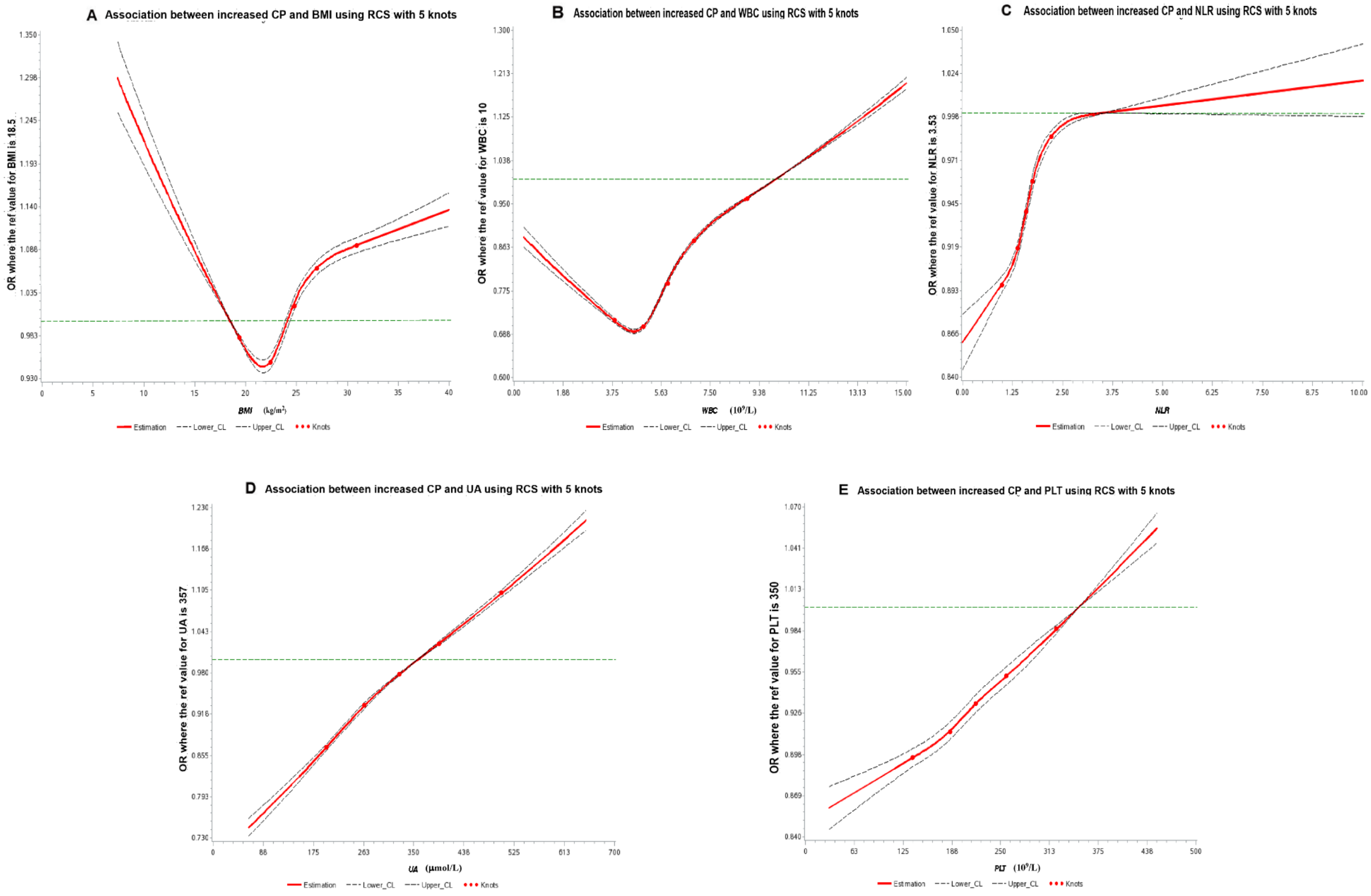

© 2024 Fu J et al. *JAMA Network Open.*

eFigure 14. The restricted cubic spline for the association between risk factors (A-E) and CP in the overall population. Solid and dashed lines represent OR and 95% CI based on restricted cubic splines in the logistic regression model. Horizontal dashed line represents the reference value. Knots were placed at the 5th, 25th, 50th, 75th and 95th percentiles of the risk factors distribution, and the reference values were listed to the left of the Y axis for all variables, e.g., the reference value of BMI as 18.5 kg/m^2. Adjustment factors were age, sex, GDP per capita, region, BMI, WBC, NLR, HR, UA, and PLT. BMI body mass index, CP carotid plaque, GDP gross domestic product, NLR neutrophil to lymphocyte ratio, PLT platelet count, UA uric acid, WBC white blood cells.

© 2024 Fu J et al. *JAMA Network Open.*
