# Supplementary material for: National and Provincial-Level Prevalence and Risk Factors of Carotid Atherosclerosis in Chinese Adults
Source: JAMA Netw Open. 2024 Jan 11;7(1):e2351225. doi: 10.1001/jamanetworkopen.2023.51225 (PMC10784858; doi:10.1001/jamanetworkopen.2023.51225)
Supplement: Supplement 2. — Data Sharing Statement [file jamanetwopen-e2351225-s002.pdf]

Data Sharing Statement

Fu. National and Provincial-Level Prevalence and Risk Factors of Carotid Atherosclerosis in Chinese Adults. *JAMA Netw Open*. Published January 11, 2024.
doi:10.1001/jamanetworkopen.2023.51225

Data
Data available: No
